# Modulation of Lanthanide Luminescence with the Mechanical Bond: Antenna‐Emitter Confinement in a Compact [2]Rotaxane

**DOI:** 10.1002/anie.202505666

**Published:** 2025-06-16

**Authors:** Anja Ramström, Daisy R. S. Pooler, Huseynagha Abasov, Monika Tomar, Stefano Crespi, Fredrik Schaufelberger

**Affiliations:** ^1^ Department of Chemistry KTH Royal Institute of Technology Teknikringen 30 Stockholm 10044 Sweden; ^2^ Department of Chemistry – Ångström Laboratory Uppsala University Uppsala Box 523, 751 20 Sweden; ^3^ Department of Chemistry University of Warwick Gibbet Hill Rd Coventry CV4 7AL UK

**Keywords:** Antenna effect, Lanthanide luminescence, Mechanical bonds, Rotaxanes, Sensing

## Abstract

Luminescent emitters based on lanthanide ions are of ubiquitous importance in the biological sciences, but typically need sensitization from a covalently attached adjacent chromophore – an “antenna” – to have suitable emission intensities. Here we show that the mechanical bond can be used to connect the antenna to the emitter, providing dynamic features and stimuli‐responsiveness to the resulting assemblies. We outline a strategy to synthesize [2]rotaxanes capped with strong chelating groups, and establish that post‐functionalization of the interlocked scaffold by lanthanide ion insertion is modular, high‐yielding and straightforward. Photophysical studies revealed effective antenna‐emitter energy transfer within the [2]rotaxane, and the sensitization mechanism as well as ring‐thread dynamics were studied with spectroscopic and computational methods. The rotaxane was shown to have high selectivity toward Cu(II) ions, acting as an efficient turn‐off sensor. This study validates the mechanical bond as a conjugation method between antennas and emitters, yielding otherwise hard‐to‐access and beneficial features to the resulting molecular systems.

## Graphical abstract

Luminescent lanthanide emitters typically need sensitization from an attached chromophore – an “antenna” – to have useful emission intensities. Here we show that the mechanical bond can be used to connect the antenna to the emitter, providing unique stimuli‐responsiveness to the resulting assemblies. The resulting lanthanide‐capped [2]rotaxanes are easy to synthesize, give good emission intensities and act as efficient metal ion sensors.

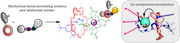

## Introduction

Mechanically interlocked molecules (MIMs) such as rotaxanes, catenanes and knots are useful for a range of applications^[^ ^1^ ^]^ including catalysis,^[^ ^2^, ^3^ ^]^ nanomedicine^[^ ^4^ ^]^ and functional materials.^[^ ^5^, ^6^ ^]^ MIMs have proven particularly efficient as molecular sensors, as the flexible binding pockets afforded by interlocking can recognize a wide range of biologically relevant cations and anions.^[^ ^7^, ^8^ ^]^ However, an area in need of further development is the readout of the MIM sensing event. The ideal sensor readout is fast, responsive, colorimetric and easily assessed (preferentially by the naked eye), as well as tuneable and cheap. While many efficient luminescent interlocked molecules have been developed, there is still much room for improvement in these areas.^[^ ^9^, ^10^, ^11^, ^12^, ^13^, ^14^, ^15^, ^16^, ^17^, ^18^ ^]^

A method that fits all criteria for ideal sensor readout is lanthanide (Ln) luminescence. The lanthanide ion series has attractive luminescence properties, namely narrow emission bands, large Stokes’ shifts, long luminescence lifetimes and resistance to photobleaching.^[^ ^19^ ^]^ Furthermore, the long lifetimes of the Ln(III) excited states lead to luminescence in the ms range, which can be exploited in combination with time‐gating methodologies to give bioimaging with low detection limits.^[^ ^20^, ^21^, ^22^ ^]^ Coordination complexes of lanthanide cations are found in many applications, particularly as cellular imaging agents and responsive bioprobes.^[^ ^23^, ^24^, ^25^, ^26^, ^27^, ^28^ ^]^ They are highly tuneable, and Ln(III) cations may be excited so that they emit in the visible (e. g., Ln═Eu and Tb) or near‐infrared (e. g., LnNd and Yb) regions of the electromagnetic spectrum. Complexes of the highly paramagnetic Gd(III) cation are also well‐established in magnetic resonance imaging (MRI), and ^177^Lu(III) is used for ultrasensitive radioimaging as well as radiotherapy.^[^ ^22^, ^26^ ^]^

**Figure 1:**
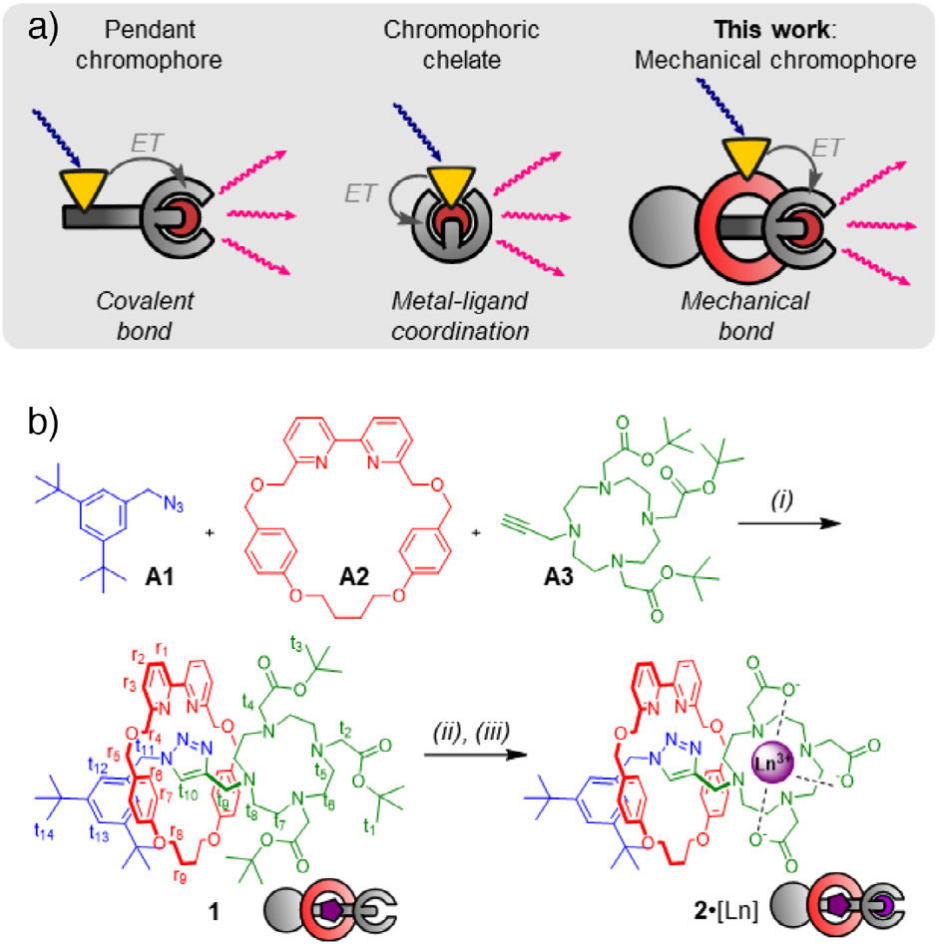
a) Cartoon overview of sensitization approaches for lanthanide luminescence. Yellow triangle = chromophore/antenna; red orb = lanthanide. Arrows added to illustrate radiative (wavy arrows) and energy transfer (gray full arrows) processes. b) Synthetic scheme. Reagents and conditions: (*i*) **A1** (1 equiv.), **A2** (1.6 equiv.), **A3** (1 equiv.), CuPF_6_•4MeCN (1.5 equiv.), N*^i^* Pr_2_Et (2 equiv.), CH_2_Cl_2_, RT, 16 h, *41%*. (*ii*) NaOH, MeOH/H_2_O, 90 °C, 16 h, *87%*. (iii) Ln(OTf)_3_ (1.1 equiv.), NEt_3_ (6 equiv.), MeOH, RT, 10 min, *90%–99%*.

However, a major challenge with applications of lanthanide luminescence is the “darkness” of the 4f–4f transition that gives rise to the emissive excited state. Since the transition is Laporte forbidden, it can only be significantly populated via intersystem crossing from a separate but closely positioned chromophore – an *antenna*.^[^ ^29^, ^30^ ^]^ As shown in Figure 1a, antennas can be classified as either pendant chromophores (i.e., covalent graft of antenna close to Ln) and chromophore chelates (i.e., direct attachment of antenna to Ln via coordination chemistry).^[^ ^30^ ^]^ This *antenna effect* is both a limitation and an opportunity for luminescent lanthanide emitters. The strict distance dependence is a limiting factor in probe design, but it can also be used to construct dynamic and switchable sensors.^[^ ^31^, ^32^, ^33^, ^34^, ^35^ ^]^

Supramolecular chemistry has previously been used to assemble or fine‐tune antenna‐emitter complexes.^[^ ^36^, ^37^, ^38^, ^39^, ^40^, ^41^, ^42^ ^]^ By harnessing non‐covalent interactions, it is possible to assemble antennas and emitters non‐covalently and to obtain distance‐dependent sensitization. The mechanical bond is an ideal tool for responsive and reversible adjustments of distance and proximity.^[^ ^1^ ^]^ Communication between the components in mechanical bonds is broadly useful to for example induce asymmetric environments for catalysis or circularly polarized luminescence,^[^ ^43^, ^44^ ^]^ but surprisingly, MIMs have barely been used in the modulation of lanthanide luminescence efficiency.^[^ ^45^ ^]^ This is largely due to the challenging synthesis, isolation and analysis of both MIMs and lanthanide chelates. Lanthanides are efficient at assembling the components of MIMs in for example rotaxanes,^[^ ^46^, ^47^ ^]^ catenanes^[^ ^48^, ^49^ ^]^ and knots,^[^ ^50^, ^51^, ^52^, ^53^ ^]^ but truly luminescent lanthanide MIMs and pseudo‐MIMs remain rare.^[^ ^54^, ^55^, ^56^ ^]^

Mechanical bonds have useful properties absent in pendant chromophores and chromophoric chelates, including co‐conformational movement, reversible steric shielding of Ln, and stimuli‐responsiveness to a broad range of inputs.^[^ ^1^ ^]^ In this study, we show that the mechanical bond can conjugate antennas to metal chelates and modulate emission of luminescent lanthanide ions (Figure 1a). We disclose the synthesis and characterization of a novel rotaxane‐based platform in which the antenna is featured within the macrocycle, and the lanthanide ion acts as a stopper (as part of a cyclen chelate). A range of trivalent lanthanide ions could be inserted in excellent yields. By intentionally targeting a compact rotaxane with a short axle we also demonstrate that the mechanical bond enforces tight intercomponent proximity, promoting luminescence sensitization. Furthermore, we utilized these new MIMs to sense transition metals,^[^ ^57^, ^58^, ^59^, ^60^ ^]^ where an Eu‐rotaxane displayed great selectivity as an “off”‐sensor toward Cu^2+^ ions.

## Results and Discussion

### Rotaxane Synthesis

**Figure 2:**
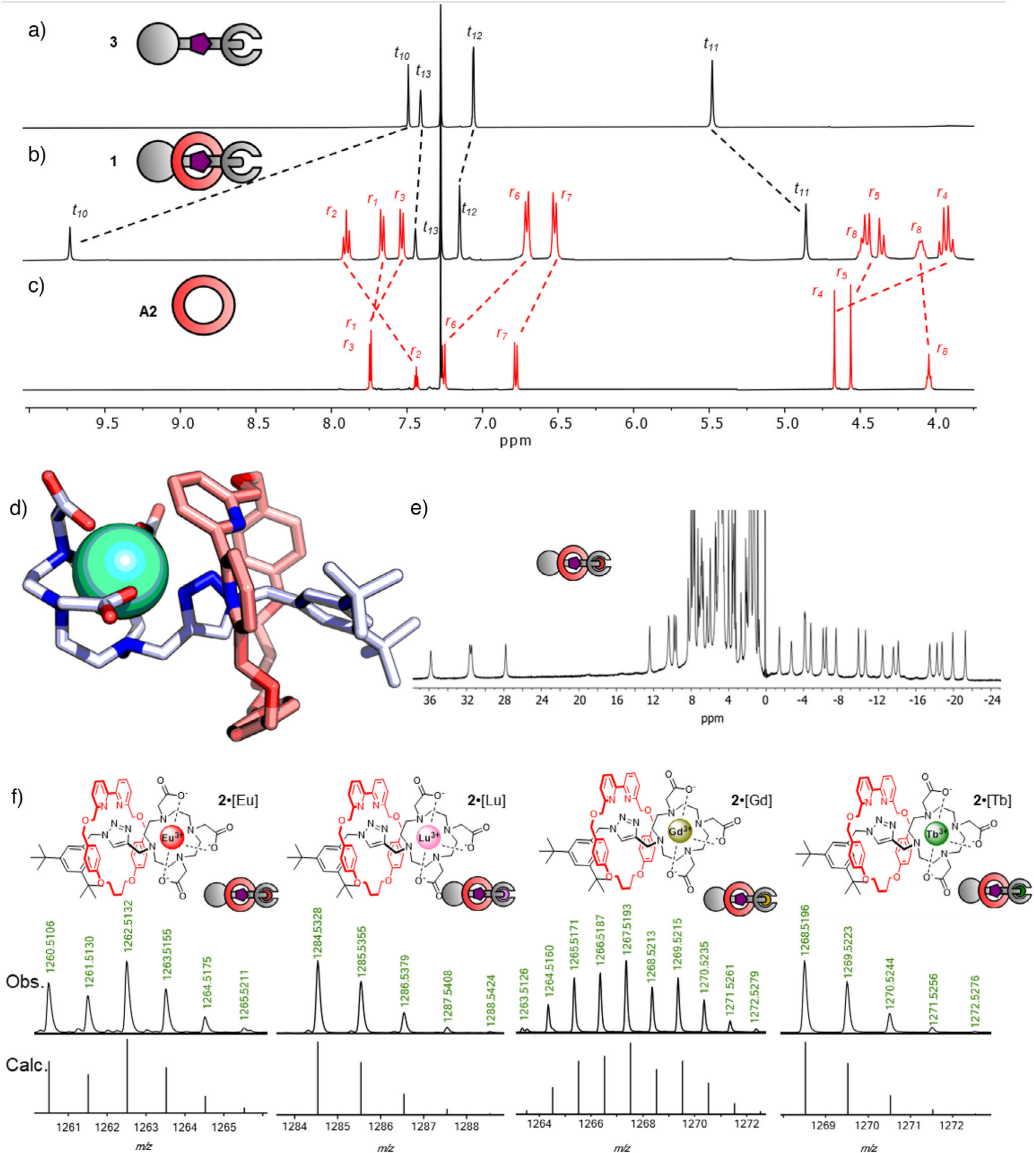
a)–c) ^1^H NMR spectra (400 MHz, 298 K, CDCl_3_) of free axle **3** (a), rotaxane **1** b) and macrocycle **A2** (c). d) DFT optimized geometry of **2**•[Eu] at the ωB97X‐D3/def2‐SVP(Eu, def2‐TZVP)/CPCM(MeOH)//GFN2‐xTB/ALPB(MeOH) level of theory. e) Paramagnetic ^1^H NMR spectrum (400 MHz, 298 K. MeOD‐*d_4_*) of **2**•[Eu] showing full range of pseudocontact shifts. f) High‐resolution mass spectrometric analysis of **2**•[Eu], **2**•[Lu], **2**•[Gd], and **2**•[Tb], with observed as well as calculated isotope distributions.

Our rotaxane assembly strategy is based on active metal template (AMT) synthesis via copper‐catalyzed azide‐alkyne cycloaddition (CuAAC).^[^ ^61^, ^62^ ^]^ AMT‐CuAAC generally requires pyridine binding motifs within the macrocycle to promote bond formation through, rather than outside, the cavity.^[^ ^63^, ^64^, ^65^, ^66^, ^67^ ^]^ We reasoned that a bipyridine (bipy) unit should be a sufficiently efficient chromophore to sensitize the rotaxane,^[^ ^68^ ^]^ given the prevalence of this motif as an antenna in the literature.^[^ ^69^, ^70^ ^]^ The bipy in this study thus served dual purposes as a Goldup‐type AMT promotor and sensitizer.^[^ ^71^ ^]^ As one of the stoppers, we used a cyclen‐derived DO3A (1,4,7,10‐tetraazacyclododecane‐1,4,7‐triacetic acid) ligand. These moieties form inert, stable chelates with lanthanides (*K*_a_ > 10^7^ M^−1^) and have been used extensively in commercial biomedical products.^[^ ^72^ ^]^ Synthesis of the building blocks **A1**‐**A3** was readily achieved as described in the supporting information (Section S4.1). A DO3A‐capped rotaxane was assembled from these building blocks using standard AMT‐CuAAC conditions (Figure 1b). We found that 1.5 equiv. of the reaction‐promoting CuPF_6_•4MeCN were required, likely to to compensate for partial complexation of the metal to the DO3A chelate. Furthermore, removing the chelated Cu ion from the DO3A proved challenging, and even extensive treatment with strong competing chelators like EDTA (ethylenediaminetetraacetic acid) did not afford adequate results. Fortunately, treatment with KCN readily removed Cu(I) through the formation of its cyanide salt. After chromatographic purification, the final rotaxane **1** was obtained in 41% isolated yield. Evidence for successful rotaxane formation was obtained from high‐resolution mass spectrometry (HRMS; observed mass for [C_74_H_106_N_9_O_10_]^+^ 1280.8058 m/z, calculated 1280.8057 m/z, Figure S42), as well as ^1^H‐ and ^13^C‐NMR spectroscopy (Figure 2, Spectra S1–S6). ^1^H‐NMR spectral analysis and comparison with separately synthesized non‐interlocked axle **3** indicated typical signal patterns for bipy‐based [2]rotaxanes made with CuAAC‐AMT synthesis (Figure 2a,c). Large resonance shifts for core protons in the axle (such as protons t_11_ and t_12_) were observed. Particularly, a sizeable downfield shift for the key triazolyl C─H resonance t_10_ (Δ*δ* = 2.2 ppm) was recorded, in line with previous data.^[^ ^17^ ^]^ For the macrocycle, expected downfield shifts for signals such as r_2_ (Δ*δ* = 0.5 ppm), and significant upfield shifts for aromatic protons such as r_6_ (Δ*δ* = 1.0 ppm) and r_7_ (Δ*δ* = 0.5 ppm) were observed. Benzylic signals r_4_ and r_5_ showed noticeable singlet‐to‐geminal‐doublets splitting_,_ due to the facial desymmetrization of the macrocycle by the unsymmetrical rotaxane axle.^[^ ^73^ ^]^

Facile cleavage of the triester *t*Bu protecting groups was achieved through NaOH mediated hydrolysis in a MeOH/H_2_O solvent mixture at 90 °C overnight, and the resulting deprotected triacid rotaxane **2**•3H was isolated in 87% yield. Note that typical DO3A deprotection conditions using strong acids are incompatible with the rotaxane, since acid induces macrocycle cleavage. ^1^H‐ and ^13^C‐NMR (Spectra S7–S11) and HRMS (Figure S43) analysis corroborated the expected structure and confirmed the integrity of the mechanical bond under the protecting group cleavage conditions. Gratifyingly, no slippage of the macrocycle from the axle was observed from this intermediate even after 3 months of storage in MeOD‐*d_4_* solution, validating the persistent interlocked conformation of the assembly (Figure S3).

Subsequent insertion of the lanthanide ion into the DO3A chelator was straightforward and could be achieved in a plug‐and‐play fashion. We optimized our protocol for insertion of Eu(III)–simple addition of Eu(OTf)_3_ with NEt_3_ in MeOH incorporated the lanthanide selectively into the DO3A unit as evidenced by liquid chromatography‐HRMS (LC trace in Figures S5 and S6, observed mass for [C_62_H_79_N_9_O_10_Eu]^+^ 1262.5132 m/z, calculated 1262.5157 m/z, see Figure 2f). The desired lanthanide‐capped rotaxane **2**•[Eu] was readily isolated and purified via trituration. To visualize how the mechanical bond could enforce proximity between the antenna and emitter, we created a model of **2**•[Eu] and carried out geometry optimization with semi‐empirical calculations followed by DFT (Figure 2d, see Supporting Information for details). The model showed the compact nature of the system and also indicated that the triazole N3 is directly coordinated to the lanthanide ion, in line with previous reports of triazole‐appended DO3A complexes.^[^ ^74^ ^]^

Rotaxane **2**•[Eu] displayed a broad NMR spectrum with significant resonance shifts due to the paramagnetic nature of Eu(III) and the fast relaxation associated with protons incorporated within its tensor environment (Figure 2e, Spectrum S12).^[^ ^75^, ^76^ ^]^ Resonances typical for Eu(III)‐shifted CH_2_ cyclen resonances (axial and equatorial) could be identified at 28,−3,−8,−12, and −15 ppm, indicative of a square antiprismatic coordination geometry in solution (as also observed in the model in Figure 2d).^[^ ^69^ ^]^ Direct comparison between **2**•[Eu] and separately synthesized Eu‐capped axle **4**•[Eu] (see Scheme S5) showed that large paramagnetic shifts occur for both the DO3A and macrocycle environments in **2**•[Eu] (Figure S7). This reflects the compact, crowded environment around the Eu(III) ion in the rotaxane, verifying the close proximity between DO3A and macrocycle.

Diversification of rotaxane **2**•3H by insertion of different lanthanide (III) ions was simple; just by changing the Ln(OTf)_3_ salt we could obtain rotaxanes **2**•[Eu], **2**•[Lu], **2**•[Gd] and **2**•[Tb] in isolated yields of 90%–99% (Figure 2f). Rotaxane **2**•[Tb] displays similar strong, sharp paramagnetic shifts as **2**•[Eu], while **2**•[Gd] has strongly broadened signals (Spectra S12–S14). For **2**•[Lu], the NMR signals are also broadened (Spectra S15) due to either DO3A chelate dynamics or macrocycle dynamics such as pirouetting.

### Photophysical Properties

**Figure 3:**
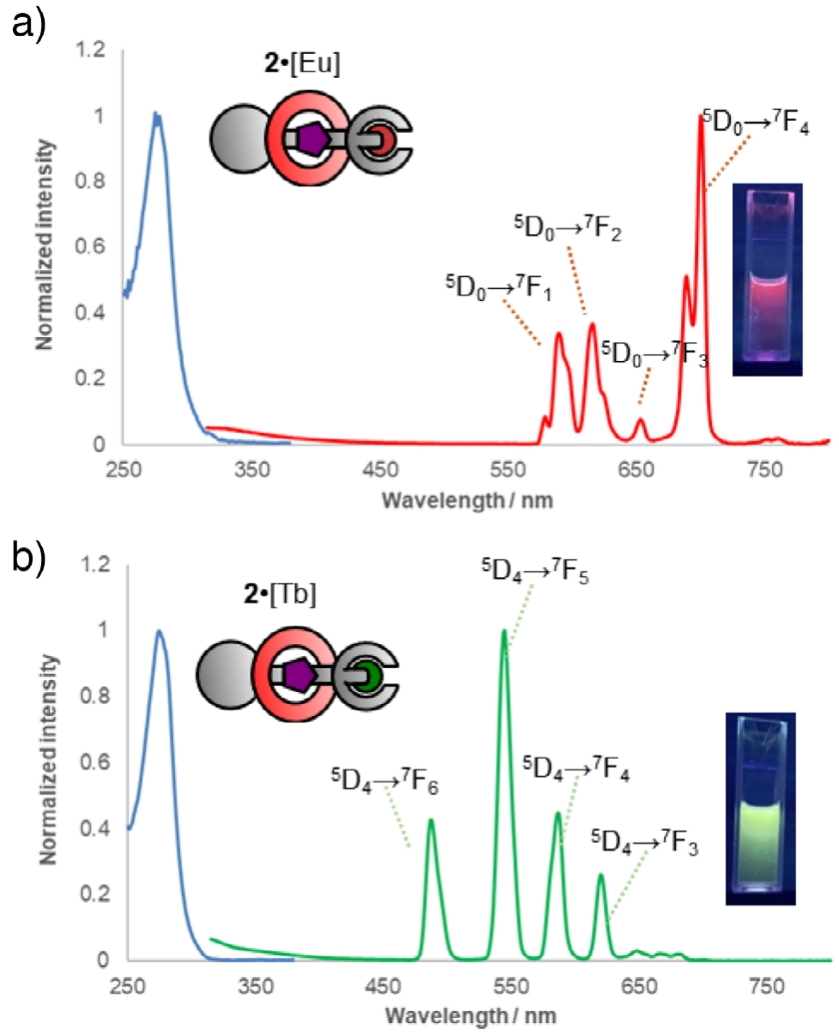
a) Normalized excitation‐emission spectroscopy analysis (10 µM, MeOH, *λ*_excitation_ = 275 nm) of **2**•[Eu] a) and **2**•[Tb] b). Inserts show photographs of the solutions under 254 nm irradiation. Excitation spectra in blue, emission spectra in red/green. Key assignable electronic transitions labelled.

**Figure 4:**
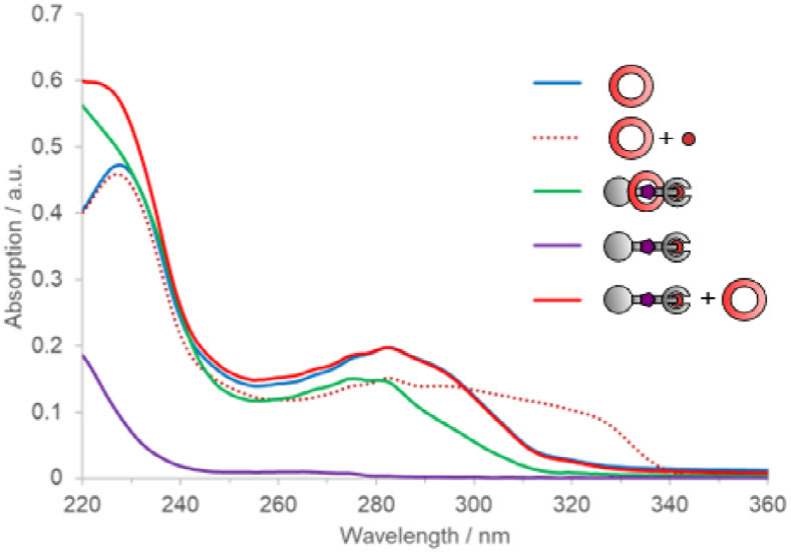
Overlaid UV–vis spectra (MeOH, 50 µM) of macrocycle **A2** (blue), **A2** + Eu(OTf)_3_ 1:1 (dotted orange), **2**•[Eu] (green), **4**•[Eu] (purple), and **4**•[Eu] + Eu(OTf)_3_ 1:1 (red).

With a range of Ln‐capped rotaxanes in hand we next explored their photophysical properties, focusing on investigating the antenna effect induced by the bipy‐furnished macrocycle. Visual inspection of **2**•[Eu] and **2**•[Tb] under 254 nm UV light showed strong luminescence, with red and green emission for Eu(III) and Tb(III), respectively. In contrast, axle **4**•[Eu] was significantly less emissive, demonstrating that the bipy sensitizing unit is necessary for strong emission. Normalized excitation‐emission spectra display the typical sharp fluorescence bands for both **2**•[Eu] and **2**•[Tb] (Figure 3a,b).^[^ ^20^, ^21^, ^22^ ^]^ The excitation maxima at 274 nm for both rotaxanes correspond to the π–π* transition in the bipy unit, confirming that the macrocycle is the primary sensitizer/antenna for these luminescent emitters (Figure 4). Irradiation at the excitation maxima for both **2**•[Eu] and **4**•[Eu] established, at minimum, a sevenfold enhancement in luminescence intensity due to sensitization from the bipy antenna (Figure S23). With irradiation at higher wavelengths (>280 nm), there is essentially no emission from free axle **4**•[Eu] (Table S4), while rotaxane **2**•[Eu] remains emissive up to ca 320 nm, as observed from the broad shoulder extending up to this wavelength in the UV–vis spectrum (Figure 4, green).

The identities of the electronic transitions for both **2**•[Eu] and **2**•[Tb]could be readily assigned based on literature data, as shown in Figure 3.^[^ ^20^, ^21^, ^22^, ^29^ ^]^ The low ^5^D_0_→^7^F_2_ and intense ^5^D_0_→^7^F_4_ transitions for **2**•[Eu] are particularly noticeable, as this type of spectral profile is characteristic of low symmetry coordination spheres.^[^ ^29^ ^]^ Time‐resolved fluorescence spectroscopy and luminescence lifetime measurements showed the expected ms range timescales for the lanthanide luminescence (~1 ms lifetime in MeOH for **2**•[Eu], Figure S13). As expected, **2**•[Gd] and **2**•[Lu] were non‐emissive.

Furthermore, we were interested in probing the mechanism of the sensitization event from our bipy antenna to the lanthanide. Lanthanide sensitization through antennas is known to occur through multiple different mechanisms, i.e., ligand‐to‐metal charge transfer (LMCT), as well as Dexter energy transfer, and (to a lesser extent) Förster resonance energy transfer.^[^ ^77^, ^78^ ^]^

LMCT requires direct coordination of the antenna to the lanthanide (*chromophore chelates*, Figure 1a), and Dexter mechanisms require a sub‐nm distance between the antenna and emitter for efficient energy transfer (ET). To distinguish LMCT from through‐space ET, we used the well‐investigated relationship between bipy coordination mode and absorption wavelength. When the bipy 2,2′‐nitrogens are protonated or donate electron density to metals via coordination, a pronounced broadening of the absorption spectra toward longer wavelengths can be observed by UV–vis spectroscopy.^[^ ^79^ ^]^ Macrocycle **A2** has an absorption maximum at 283 nm with low absorption beyond 315 nm (Figure 4, blue line). In contrast, when Eu(III) is coordinated into the macrocycle, the absorption band redshifts until 339 nm (dotted orange line).^[^ ^80^ ^]^ However, rotaxane **2**•[Eu]–with the Eu(III) coordinated to DO3A–exhibits a similar absorption spectrum to free macrocycle **A2**, indicating limited intramolecular coordination of the bipy nitrogen atoms to the Eu center (green line). This data strongly indicates that the conformational restrictions imposed on the bipy ligand by the interlocking prohibit dative bond formation to the lanthanide. Similarly, adding macrocycle **A2** to axle **4**•[Eu] did not produce appreciable coordination (red line). Measurement of the number of solvent‐accessible coordination sites (for MeOH/MeOD) in **2**•[Eu] and **4**•[Eu] via Horrocks’ method corroborated these observations (see further Section S5.5.4).^[^ ^81^ ^]^ The rotaxane had a q value of 1.1, while the axle gave a value of 1.2, with both measurements (within error) showing that one free coordination site of the Ln is available for coordination by solvent molecules.

### Conformational Analysis

The mechanical bond provides an efficient way to engineer constrained dynamics between the antenna and emitter. To explore this further, we used density functional theory (DFT) to study the impact of the mechanical bond on the coordination of the Ln(III) ions and its influence on through‐space sensitization. We conducted a theoretical investigation of **2**•[Eu] to study the interaction of its antenna (bipy macrocycle) and emitter (Eu‐DO3A). Initial geometry optimization was carried out using Grimme's semi‐empirical quantum mechanical (SQM) methods, which provide reliable geometries for lanthanide complexes at a reduced computational cost.^[^ ^82^ ^]^ We opted for GFN2‐xTB^[^ ^83^ ^]^ using MeOH as implicit solvent (ALPB solvation model)^[^ ^84^ ^]^ to reflect experimental conditions. Due to the high degree of flexibility and dynamic nature of the rotaxane, conformational sampling using CREST^[^ ^85^ ^]^ was carried out at the GFN2‐xTB/ALPB(MeOH) level of theory revealing multiple stable co‐conformations (see Table S7). Further geometry optimization and frequency calculations were performed using density functional theory (DFT) at the ωB97X‐D3/def2‐SVP(Eu, def2‐TZVP) level, using the conductor‐like polarizable continuum (CPCM) model for implicit MeOH solvation (SI Section S6).^[^ ^86^, ^87^, ^88^ ^]^ Our DFT calculations confirm the close spatial distance between the DO3A‐Ln and the bipy macrocycle components (Figure 2d, Section S6.1). The Ln resides in a distorted square antiprismatic coordination geometry with the triazole directly coordinated, and with a close association to the pyridine heterocycles. The lanthanide is essentially completely protected by the ligand, with the macrocycle shielding the open coordination sphere (Figure S41).

**Figure 5:**
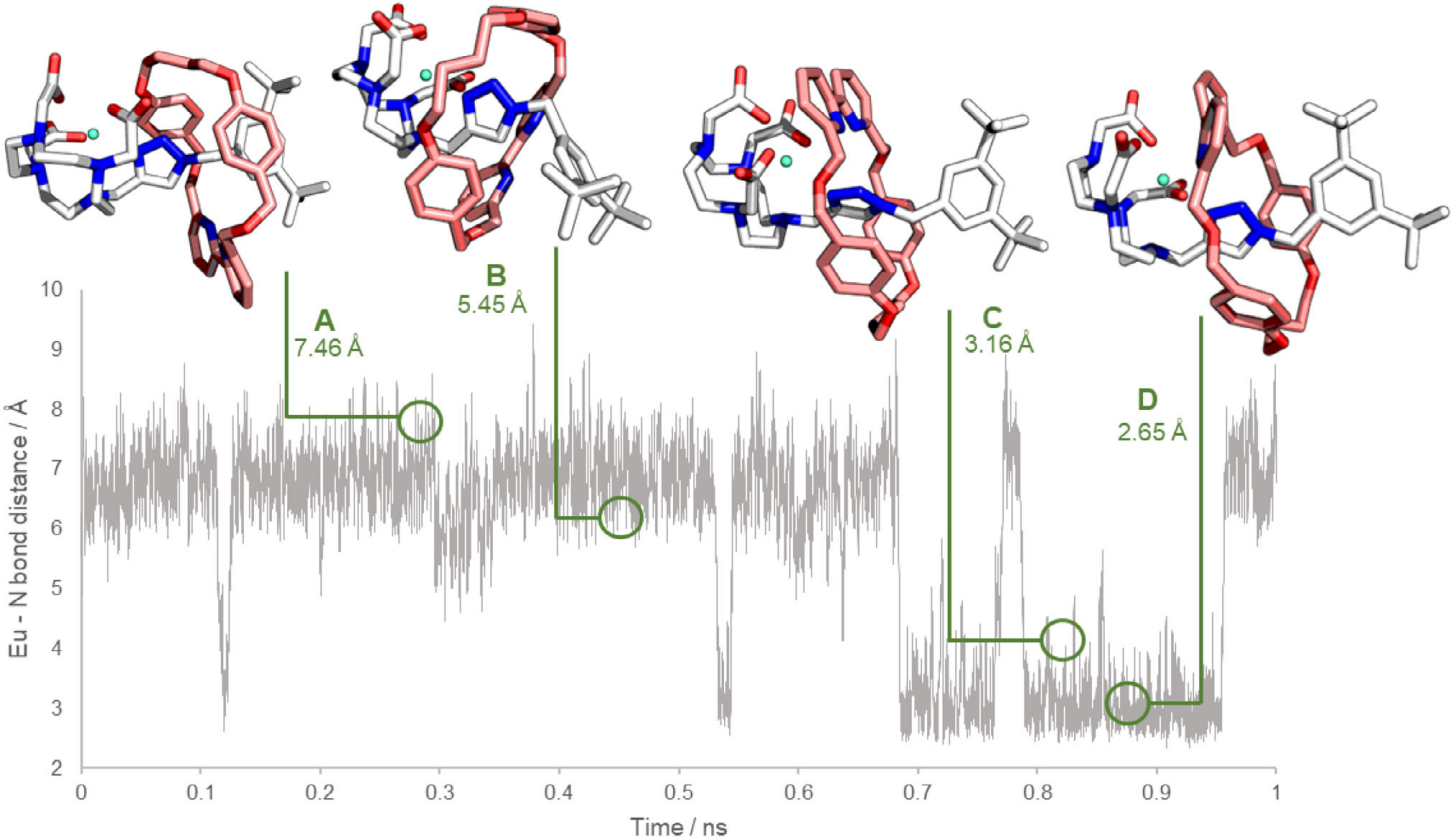
Co‐conformational dynamics of ring‐thread components in **2**•[Eu] as investigated via metadynamics (GFN2‐xTB/ALPB(MeOH)). Distance is given between one bipyridine N‐atom and the Eu center as a function of simulation runtime. A‐D are representative molecular conformations for each of the populated states.

Furthermore, we conducted metadynamics^[^ ^89^ ^]^ simulations at the GFN2‐xTB/ALPB(MeOH) level (Figure 5) to probe the ground state potential energy surface of **2**•[Eu], revealing possible co‐conformations of the bipy antenna and Eu(III)‐DO3A emitter within our compact rotaxane. Studies over a 1.0 ns time regime clearly demonstrate that the short axle limits the conformational space available for the bipy macrocycle, allowing only pirouetting of the macrocycle with effectively no translational shuttling. This means the sensitizer is confined within the Dexter radius of the lanthanide^[^ ^78^ ^]^ at all times (see Figure 5 and Supplementary GIF 1). Two preferred co‐conformational regimes could be observed, with the bipy essentially being in either distant (conformers A‐─B, N─Eu distance ~7 Å) or close to (conformers C‐D, NEu distance −3 Å) the open Ln coordination site (roughly corresponding to the 180° pirouetting of the macrocycle around the axis). Metadynamics simulations were also carried out with explicit methanol solvation using Grimme's Quantum Cluster Growth (QCG)^[^ ^90^ ^]^ solvation model, and no significant differences were observed between the co‐conformations in each case. The previously conducted conformational sampling using CREST and comparison of the energies of conformers <6 kcal mol^−1^ away from the global minimum indicated relatively low energy differences between the co‐conformers, with the close‐alignment conformation being slightly favored (Table S7). The metadynamics trajectory captures the stochastic pirouetting motion of the bipy macrocycle, while confirming that the macrocycle has rapid dynamics on the same timescales as ligand coordination events to the Ln(III) center. These simulations explain both the sensitizing effect of the bipy and the measured q values. We see sufficient confinement for consistent co‐component energy transfer, yet at the same time, the macrocycle is dynamic enough to accommodate ligand exchange. This is a feature of the mechanically interlocked system not easily replicated with covalently grafted system.

### Cation Sensing

**Figure 6:**
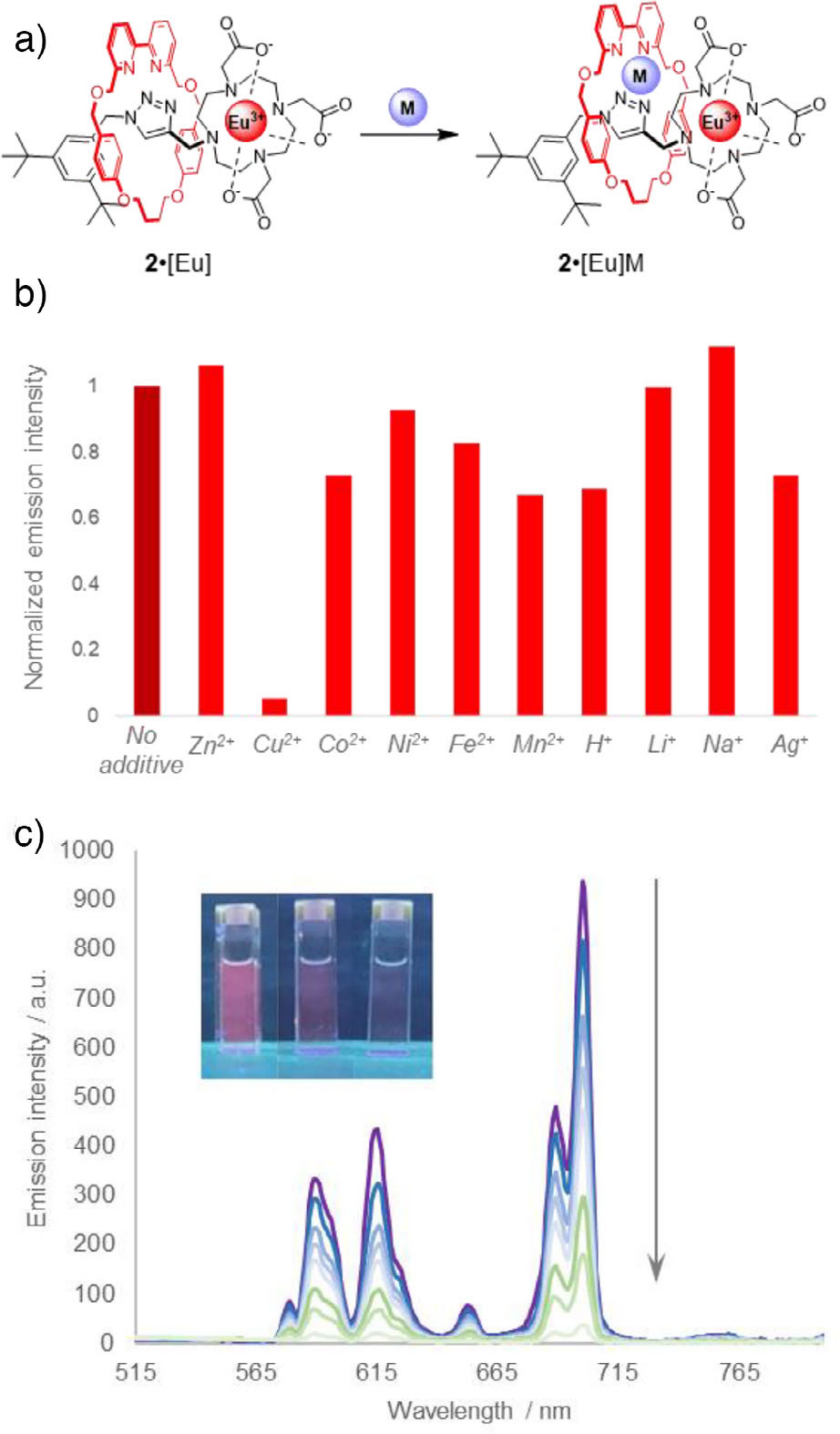
Sensing of cations with luminescent readout. a) Hypothesized intracavity binding mode for cations via the bipy moiety (exact nature of binding not known). b) Effect of metal ion addition on luminescence emission intensity for **2**•[Eu] (10 µM, MeOH). Emission measured at 700 nm and normalized versus sample without cation additive. 5 equiv. of each metal ion added, and 1 equiv. of H^+^. c) Changes in Eu(III) emission upon titration with increasing amounts of Cu(II) (up to 5 equiv., *λ*_excitation_ = 275 nm, see Supporting Information for details).

Both rotaxanes and lanthanide complexes have previously been studied in sensing applications (Figure 6a).^[^ ^7^, ^8^, ^23^, ^24^, ^25^, ^26^, ^27^, ^28^ ^]^ Furthermore, bipy rotaxanes have been shown to be excellent ligands for transition metals, as cavity size and nature of the axle can be tailored to achieve selectivity for specific metal binding.^[^ ^91^ ^]^ We were interested to see if the constrained binding pocket inside the Ln‐tagged rotaxanes would enable increased selectivity for cation sensing, especially considering previous reports of Ln‐complexes where metal coordination to bipyridine and phenanthroline sensitizers could modulate lanthanide luminescence.^[^ ^69^, ^70^ ^]^

Upon addition of a series of transition and alkali metals to **2**•[Eu], we discovered a strong OFF‐response to Cu(II) ions (Figure 6b, Table S5). Addition of 5 equiv. of Cu(ClO_4_)_2_ quenched > 95% luminescence intensity (Figure 6c). All other tested cations (as well as protonation) yielded low to no response in emission quenching, meaning excellent selectivity for Cu(II) was obtained within this dataset. By measuring the UV‐Vis spectra of **2**•[Eu] after addition of metals or acid, we found that only a few metals (Cu^2+^, Fe^2+^, and Ag^+^) showed any binding at all with the bipy nitrogens (Figures S25–S28), indicating that the rotaxane is imposing stricter selection rules for binding than typically observed for a simple free bipy unit. Previous observations of Cu^2+^‐based modulation of europium luminescence attribute this phenomenon to dynamic quenching of the lanthanide excited state due to the overlap between the Eu^3+^ emission bands and Cu^2+^ d─d absorption bands.^[^ ^92^, ^93^ ^]^

Particularly remarkable here is the sensing selectivity for Cu(II) over Fe(II) and Co(II), which act as efficient quenchers in covalently phenanthroline‐tagged Ln emitters.^[^ ^69^ ^]^ This result showcases properties of the mechanical bond not achievable with comparable covalent linkages.

The precise mode of binding of transition metals to **2**•[Eu] is not known at this point, though we note that we could not observe cation complexes via ESI‐HRMS, and metal addition also did not lead to a change in q value (Table S6). Further investigations to understand the observed selectivity are ongoing in our laboratory.

## Conclusion

Here we have demonstrated that the mechanical bond can be used to induce and modulate the luminescence emission from lanthanide (III) ions. Using active metal template synthesis, we have prepared a compact rotaxane scaffold with a sensitizing bipyridine antenna in the macrocycle and a cyclen‐based stopper into which lanthanide (III) ions can be inserted post‐assembly. Photophysical measurements confirmed the sensitizing role of the macrocyclic component and the through‐space energy transfer between mechanical bond components, while mechanistic and computational studies revealed interesting dynamic behavior where the ring shields the lanthanide without actively coordinating with the metal center. Furthermore, the rotaxanes functioned as transition metal sensors with exquisite Cu(II) selectivity, demonstrating how the mechanical bond can improve sensing selectivity compared to covalently grafted systems.

This study demonstrates that the mechanical bond can be considered a third way of connecting antennas to lanthanide emitters next to covalent grafts and direct coordination. As the mechanical bond has significant dynamic movement, assembling an antenna‐emitter system like this enables entirely new ways to construct bioresponsive probes, sensors and imaging agents. We believe these scaffolds are of interest both for developing new classes of multiplexed diagnostic devices and for advanced applications in responsive magnetic resonance imaging contrast agents or radiotracers.

## Supporting Information

Experimental procedures, additional control experiments, luminescence data, UV–vis spectroscopy, NMR analysis and mass spectra (PDF). Optimized geometries as xyz coordinates (zip) and metadynamics trajectories (mp4). The authors have cited additional references within the Supporting Information.^[^ ^93^, ^94^, ^96^, ^97^, ^98^, ^99^, ^100^, ^101^, ^102^, ^103^, ^104^ ^]^

## Conflict of Interests

The authors declare no conflict of interest.

## Supporting information



Supporting Information

Supporting Information

Supporting Information

## Data Availability

The data that support the findings of this study are available in the Supporting Information of this article.
