# Supplementary material for: Modulation of Lanthanide Luminescence with the Mechanical Bond: Antenna‐Emitter Confinement in a Compact [2]Rotaxane
Source: Angew Chem Int Ed Engl. 2025 Jun 16;64(32):e202505666. doi: 10.1002/anie.202505666 (PMC12322636; doi:10.1002/anie.202505666)
Supplement: Supplementary file 1 — Supporting Information [file ANIE-64-e202505666-s003.docx]

**Modulation of Lanthanide Luminescence with the Mechanical Bond: Antenna-Emitter Confinement in a Compact [2]Rotaxane**

Anja Ramström^1^, Daisy R. S. Pooler^1^, Huseynagha Abasov^1^, Monika Tomar^2^, Stefano Crespi^2^ and Fredrik Schaufelberger* ^1,3^

1. KTH Royal Institute of Technology, Department of Chemistry, Teknikringen 30, 10044 Stockholm, Sweden

2. Uppsala University, Department of Chemistry - Ångström Laboratory, Box 523, 751 20 Uppsala, Sweden.

3. University of Warwick, Department of Chemistry, Gibbet Hill Road, Coventry CV4 7AL, UK

*E-mail: Fredrik.Schaufelberger@warwick.ac.uk

-Supporting Information-

Table of Contents

[S1. ABBREVIATIONS 3](#_Toc197443441)

[S2. GENERAL EXPERIMENTAL 4](#_Toc197443442)

[S3. REACTIONS SCHEMES 6](#_Toc197443443)

[S3.1. Synthesis of Azide Stopper **A1** 6](#_Toc197443444)

[S3.2. Synthesis of Macrocycle **A2** 6](#_Toc197443445)

[S3.3. Synthesis of DO3A Stopper **A3** 6](#_Toc197443446)

[S3.4. Synthesis of Rotaxane **2**•[Ln] 7](#_Toc197443447)

[S3.5. Synthesis of Axle **4**•[Eu] 7](#_Toc197443448)

[S4. EXPERIMENTAL PROCEDURES 8](#_Toc197443449)

[S4.1 Synthetic Procedures and Characterization Details 8](#_Toc197443450)

[S4.2. Further Experimental Procedures 14](#_Toc197443451)

[S5. SUPPLEMENTARY DATA 15](#_Toc197443452)

[S5.1. Screening of Conditions for AMT-CuAAC Rotaxane Formation 15](#_Toc197443453)

[S5.2. Stability Study of Rotaxane 18](#_Toc197443454)

[S5.3. Protonation of DO3A-containing Compounds 19](#_Toc197443455)

[S5.4. Lanthanide Coordination 20](#_Toc197443456)

[S5.5. Photophysical Characterization 22](#_Toc197443457)

[S5.6. Emission Amplification via Intercomponent Energy Transfer 31](#_Toc197443458)

[S5.7. Metal Addition to **2**•[Eu] 33](#_Toc197443459)

[S6. COMPUTATIONAL DETAILS 44](#_Toc197443460)

[S6.1. Geometry Optimization 44](#_Toc197443461)

[S6.2. Conformational Sampling 44](#_Toc197443462)

[S6.3. Metadynamics Simulations 45](#_Toc197443463)

[S6.4. Space-filling Models of **2**•[Eu] 46](#_Toc197443464)

[S6.5. Explicit solvation of **2**•[Eu] using Quantum Cluster Growth 47](#_Toc197443465)

[S7. MASS SPECTRA 48](#_Toc197443466)

[S8. NMR SPECTRA 53](#_Toc197443467)

[S9. REFERENCES 67](#_Toc197443468)

# S1. ABBREVIATIONS

Abbreviations: AMT, Active Metal Template; bipy, bipyridine; DCM, dichloromethane; DMF, *N*,*N*-dimethylformamide; DMSO, dimethylsulfoxide; DO3A, tetraazacyclododecane-triacetic acid; DOTA, tetraazacyclododecane-tetraacetic acid; Ln, lanthanide; MeCN, acetonitrile; MeOH, methanol; o.n., overnight; OTf, trifluoromethane sulfonate; PE, Petroleum ether; RT, room temperature; TLC, thin layer chromatography.

# S2. GENERAL EXPERIMENTAL

Unless stated otherwise, reagents were obtained from commercial sources and used without further purification. Reactions were carried out in anhydrous solvents and under an N_2_ atmosphere. Anhydrous solvents were obtained by passing the solvent through an activated alumina column in a Glass Contour solvent dispensing system and stored over molecular sieves. Compounds **A1**^94^, **S6**^95^, **A3**^96^ and **S1-S5+A2**^71^ were synthesized as previously described. ^1^H NMR spectra were recorded on a Bruker Avance DMX 500 MHz NMR spectrometer and a Bruker Ascend 400 spectrometer (400 MHz). Chemical shifts are reported in parts per million (ppm) from high to low frequency using the residual solvent peak as the internal reference (CDCl_3_ = 7.26 ppm, CD_3_OD = 3.31 ppm, and CD_3_CN = 1.94 ppm. All ^1^H resonances are reported to the nearest 0.01 ppm. The multiplicity of ^1^H signals are indicated as: s = singlet; d = doublet; t = triplet; q = quartet; m = multiplet; br = broad; app = apparent; or combinations of thereof. Coupling constants (*J*) are quoted in Hz and reported to the nearest 0.1 Hz. Where appropriate, averages of the signals from peaks displaying multiplicity were used to calculate the value of the coupling constant. ^13^C NMR spectra were recorded on the same spectrometers with the central resonance of the solvent peak as the internal reference (CD_3_CN = 118.26 ppm, CDCl_3_ = 77.16 ppm and CD_3_OD = 49.00 ppm. All ^13^C resonances are reported to the nearest 0.01 ppm. For new compounds, DEPT, COSY, HMBC and HSQC experiments were used to aid spectral assignment. Fully characterized compounds were chromatographically homogeneous except where indicated otherwise in experimental descriptions.

Flash column chromatography was carried out using Silica 60 Å (particle size 40–63 μm, Merck, Sweden) as the stationary phase. Size exclusion chromatography was carried out using Biobeads S-X3 or Sephadex LH-20 support beads as the stationary phase. TLC was performed on precoated silica gel plates (0.25 mm thick, 60 F_254_, Merck, Germany) and visualized using both short and long wave ultraviolet light in combination with standard laboratory stains (basic potassium permanganate). Liquid-chromatography high-resolution mass spectrometry was performed on a LC-MS-QTOF 6530C instrument (Agilent) with an analytical ZORBAX RRHD Extend-C18 column, 2.1 x 100 mm, 1.8 µm (Agilent).

All optical spectroscopy measurements were conducted in MeOH unless otherwise specified. Quartz cells with a 1 cm optical path length were utilized for room temperature measurements. The steady-state emission and excitation spectra, Eu(III) luminescence lifetimes, and time-resolved emission and excitation spectra on the µs-ms timescale were obtained using a Horiba FluoroMax-4P instrument. Emissions were corrected for wavelength sensitivity using the spectrometer's correction function. All measurements were performed at room temperature unless noted otherwise. Lifetimes were recorded 0.05 ms after pulsed excitation at the ligand's excitation maxima (λ_ex_) by monitoring the decay of the primary lanthanide emission peaks (Eu(III): 616/700 nm; Tb(III): 545 nm). The time increments following the initial delay were adjusted between 0.2–20 μs, depending on the lifetime, to ensure adequate sampling of the decay. The resulting data was fitted using single and double exponential decay models in OriginPro 9, with the most reliable values determined based on the adjusted R² value and residuals' shape. A typical relative error of 10% was observed across repeated measurements of the same sample. For photophysical measurements on **4**•[Eu] (and comparison to **2**•[Eu]) an Edinburgh Instruments FS5 spectrofluorometer was used and each measurement was repeated 5 times.

# S3. REACTIONS SCHEMES

## S3.1. Synthesis of Azide Stopper A1

Scheme S1. Synthesis of azide stopper A1.

## S3.2. Synthesis of Macrocycle A2

Scheme S2. Synthesis of macrocycle A2.

## S3.3. Synthesis of DO3A Stopper A3

Scheme S3. Synthesis of DO3A stopper A3.

## S3.4. Synthesis of Rotaxane 2•[Ln]

Scheme S4. Synthesis of rotaxane 2•[Ln].

## S3.5. Synthesis of Axle 4•[Eu]

Scheme S5. Synthesis of thread 4•[Eu].

# S4. EXPERIMENTAL PROCEDURES

## S4.1 Synthetic Procedures and Characterization Details

**1**N*^i^*Pr_2_Et (35 µL, 0.20 mmol, 2 equiv.) was added to a solution of **A1** (25 mg, 0.10 mmol, 1.0 equiv.), **A2** (77 mg, 0.16 mmol, 1.6 equiv.), **A3** (55 mg, 0.10 mmol, 1.0 equiv.) and [Cu(MeCN)_4_]PF_6_ (56 mg, 0.15 mmol, 1.5 equiv.) in degassed, dry CH_2_Cl_2_ (8 mL). The mixture was stirred under an N_2_ atmosphere at RT overnight. To this reaction mixture, MeOH (20 mL) followed by a solution of KCN (39 mg, 0.60 mmol, 6.0 eq.) in MeOH (4 mL) was added and stirred vigorously for 1 h. Thereafter, the mixture was concentrated under reduced pressure, redissolved in CH_2_Cl_2_ (25 mL) and washed with brine (25 mL). The aqueous phase was extracted with additional CH_2_Cl_2_ (2 x 25 mL), whereafter the combined organic phase was washed with brine, dried with MgSO_4_ and concentrated under reduced pressure. The resulting crude was run through a neutral Al_2_O_3_ plug (9:1 CH_2_Cl_2_/EtOAc) and SEC column (CH_2_Cl_2_, Biobeads S-X3) to remove excess macrocycle. Final purification by SiO_2_ column chromatography (100:100:0.5 CH_2_Cl_2_/PE/EtOH with MeCN gradient from 6% – 20%) yielded **1** as a colorless solid (52.5 mg, 41 %) with a purity level high enough for further experiments (5-10% impurities). For NMR characterization, the product was protonated with HCl in MeOH, see section S5.3.

**^1^H NMR** (500 MHz, CDCl_3_) δ 9.71 (s, 1H, H_5_), 7.88 (t, *J* = 7.8 Hz, 2H, H_b_), 7.65 (d, *J* = 7.8 Hz, 2H, H_a_), 7.52 (d, *J* = 7.7 Hz, 2H, H_c_), 7.43 (t, *J* = 1.7 Hz, 1H, H_1_), 7.14 (d, *J* = 1.8 Hz, 2H, H_2_), 6.69 (d, *J* = 8.1 Hz, 4H, H_f_), 6.51 (d, *J* = 8.1 Hz, 4H, H_g_), 4.85 (s, 2H, H_4_), 4.51 – 4.40 (m, 4H, H_e,h_), 4.34 (d, *J* = 11.7 Hz, 2H, H_e_), 4.08 (dd, *J* = 12.5, 6.2 Hz, 2H, H_h_), 3.92 (q, *J* = 11.9 Hz, 4H, H_d_), 3.52 (s, 2H, H_6_), 3.26 (s, 2H, H_12_), 3.14 (s, 4H, H_11_), 2.93 – 2.21 (m, 16H, H_7-10_), 2.21 – 2.14 (m, 2H, H_i_), 2.03 – 1.92 (m, 2H, H_i_), 1.47 (s, 18H, H_13_), 1.46 (s, 9H, H_14_), 1.25 (s, 18H, H_3_); **^13^C NMR** (126 MHz, CDCl_3_) δ 170.91, 170.40, 159.62, 158.69, 156.73, 151.46, 138.15, 134.00, 132.70, 129.92, 129.41, 128.25, 123.93, 123.61, 122.27, 121.96, 114.83, 81.93, 81.79, 73.63, 71.30, 66.51, 57.44, 54.89, 54.59, 53.95, 51.76, 50.07, 48.39, 46.54, 34.93, 31.50, 28.33, 28.25, 24.92; **HRMS** (ESI^+^): Calcd. for C_74_H_105_O_10_N_9_H^+^: 1280.8057, found 1280.8058 [M+H]^+^.

**2**•**3H**1 M NaOH (aq.) (1.6 mL, 1.6 mmol, 43 equiv.) was added to a solution of **1** (46.5 mg, 0.036 mmol, 1 equiv.) in MeOH (6.2 mL) and the mixture was left at 90 °C in a pressure tube overnight. The MeOH was removed at reduced pressure, the residue was diluted with water (5.0 mL), the pH was adjusted until slightly acidic by slow addition of 1 M HCl (aq.). The solid was filtered off, washed with water, dissolved in MeOH and dried under reduced pressure. This yielded **2**•3H as a colorless solid (36.4 mg, 87 %).

**^1^H NMR** (500 MHz, CD_3_OD) δ 9.67 (s, 1H, H_5_), 7.94 (t, *J* = 7.8 Hz, 2H, H_b_), 7.83 (d, *J* = 7.8 Hz 2H, H_a_), 7.56 (d, *J* = 7.7 Hz, 2H, H_c_), 7.49 (t, *J* = 1.8 Hz, 1H, H_1_), 7.30 (d, *J* = 1.8 Hz, 2H, H_2_), 6.79 (d, *J* = 8.4 Hz, 4H, H_f_), 6.67 (d, *J* = 8.5 Hz, 4H, H_g_), 5.27 (s, 2H, H_4_), 4.73 – 4.64 (m, 2H, H_h_), 4.57 (d, *J* = 11.7 Hz, 2H, H_e_), 4.26 (d, *J* = 11.7 Hz, 2H, H_e_), 4.11 – 4.04 (m, 2H, H_h_), 4.00 (d, *J* = 11.8 Hz, 2H, H_d_), 3.87 (d, *J* = 11.8 Hz, 2H, H_d_), 3.70 (s, 2H, H_12_), 3.37 (s, 2H, H_6_), 3.30 – 3.19 (m, 6H, H_7-10_), 3.18 – 3.06 (m, 2H, H_7-10_), 2.98 (s, 4H, H_11_), 2.80 – 2.50 (m, 6H, H_7-10_), 2.50 – 2.33 (m, 2H, H_7-10_), 2.21 – 2.11 (m, 2H, H_i_), 2.05 – 1.94 (m, 2H, H_i_), 1.23 (s, 18H, H_3_); **^13^C NMR** (126 MHz, CD_3_OD) δ 174.24, 170.44, 160.83, 160.07, 157.76, 152.74, 139.43, 135.55, 135.03, 131.18, 130.86, 129.75, 124.92, 124.70, 123.51, 123.17, 116.07, 74.32, 72.28, 67.59, 56.67, 55.96, 55.71, 52.44, 50.42, 35.71, 31.83, 25.95. Four ^13^C signals are overlapping with the MeOD peak.; **HRMS** (ESI^+^): Calcd. for C_62_H_81_O_10_N_9_H^+^: 1112.6179, found 1112.6118 [M+H]^+^.

**General procedure for lanthanide ion complexation:**

Et_3_N (6.0 equiv.) was added to a solution of **2**•3H (1.0 equiv.) and Ln(OTf)_3_ (1.1 equiv.) in MeOH and left to stir at RT for 10 min.^[[1]](#footnote-2)^ The solvent was removed under reduced pressure and the solid was triturated three times with water to remove HEt_3_NCl and excess Ln(OTf)_3_. Drying the remaining solid under reduced pressure yielded **2**•[Ln].

**2•[Eu]
2**•[Eu] was prepared according to the general procedure using **2**•3H (2.6 mg, 2.3 µmol, 1.0 equiv.), Eu(OTf)_3_ (1.5 mg, 2.5 µmol, 1.1 equiv.), Et_3_N (1.9 µL, 14 µmol, 6.0 equiv.) and MeOH (2 mL). This yielded **2**•[Eu] as a colorless solid (2.9 mg, >99 %).

**^1^H NMR** (500 MHz, CD_3_OD) δ 35.82, 31.64, 31.46, 27.81, 12.43, 10.41, 9.82, 9.62, 8.33, 8.02, 7.83, 7.64, 7.28, 7.14, 6.98, 6.88, 6.73, 6.32, 5.98, 5.43, 4.63, 4.01, 3.47, 2.68, 2.19, 2.03, -1.42, -2.69, -4.10, -4.18, -4.77, -6.09, -6.43, -7.47, -9.87, -10.59, -12.42, -13.59, -14.12, -17.47, -18.25, -18.76, -19.92, -21.27; **HRMS** (ESI^+^): Calcd. for C_62_H_78_O_10_N_9_EuH^+^: 1262.5157, found 1262.5177 [M+H]^+^.

**2•[Lu]
2**•[Lu] was prepared according to the general procedure using **2**•3H (2.6 mg, 2.3 µmol, 1.0 equiv.), Lu(OTf)_3_ (1.6 mg, 2.5 µmol, 1.1 equiv.), Et_3_N (1.9 µL, 14 µmol, 6.0 equiv.) and MeOH (2 mL). This yielded **2**•[Lu] as a colorless solid (2.6 mg, 90 %). ^1^H-NMR spectra were broadened due to internal dynamics.

**HRMS** (ESI^+^): Calcd. for C_62_H_78_O_10_N_9_LuH^+^: 1284.5352, found 1284.5328 [M+H]^+^.

**2•[Gd]
2**•[Gd] was prepared according to the general procedure using **2**•3H (2.7 mg, 2.3 µmol, 1.0 equiv.), Gd(OTf)_3_ (1.6 mg, 2.6 µmol, 1.1 equiv.), Et_3_N (1.9 µL, 14 µmol, 6.0 equiv.) and MeOH (2 mL). This yielded **2**•[Gd] as a colorless solid (2.9 mg, >99 %). ^1^H-NMR spectra were severely broadened due to paramagnetism.

**HRMS** (ESI^+^): Calcd. for C_62_H_78_O_10_N_9_GdH^+^: 1267.5185, found 1267.5193 [M+H]^+^.

**2•[Tb]
2**•[Tb] was prepared according to the general procedure using **2**•3H (3.6 mg, 3.2 µmol, 1.0 equiv.), Tb(OTf)_3_ (2.1 mg, 3.5 µmol, 1.1 equiv.), Et_3_N (2.7 µL, 19 µmol, 6.0 equiv.) and MeOH (2 mL). This yielded **2**•[Gd] as a colorless solid (3.9 mg, 96 %).

**^1^H NMR** (400 MHz, CD_3_OD) δ 263.45, 245.77, 218.61, 215.85, 53.42, 49.22, 45.33, 25.21, 24.95, 20.61, 16.47, 9.47, 8.93, 6.50, 6.28, 4.49, 3.68, -0.74, -3.08, -3.78, -4.57, -6.38, -7.93, -20.01, -25.85, -32.29, -33.64, -48.42, -55.51, -59.36, -63.35, -64.30, -70.44, -73.04, -75.24, -81.43, -106.40, -115.34, -130.96, -147.55, -208.12, -367.26; **HRMS** (ESI^+^): Calcd. for C_62_H_78_O_10_N_9_TbH^+^: 1268.5198, found 1268.5196 [M+H]^+^.

**3**A solution of **A1** (24.4 mg, 0.10 mmol, 1.0 equiv.), **A3** (55.8 mg, 0.10 mmol, 1.0 equiv.) and [Cu(MeCN)_4_]PF_6_ (82.6 mg, 0.22 mmol, 2.2 equiv.) in degassed CH_2_Cl_2_ (8 mL) was stirred under N_2_ atmosphere overnight. To this reaction mixture, MeOH (26 mL) was first added, followed by a solution of KCN (57 mg, 0.88 mmol, 8.8 equiv.) in MeOH (5.9 mL). The mixture was stirred vigorously for 1 h. Thereafter, the solution was concentrated under reduced pressure, redissolved in CH_2_Cl_2_ (25 mL) and washed with brine (25 mL). The aqueous phase was extracted with additional CH_2_Cl_2_ (2 x 25 mL), whereafter the combined organic phase was washed with brine, dried with MgSO_4_ and concentrated under reduced pressure. Purification of the resulting crude by Al_2_O_3_ column chromatography (8:2 CH_2_Cl_2_/EtOAc), yielded **3** as a colorless solid (19 mg, 24 %).

**^1^H NMR** (500 MHz, CDCl_3_) δ 7.47 (s, 1H, H_5_), 7.40 (t, *J* = 1.8 Hz, 1H, H_1_), 7.04 (d, *J* = 1.8 Hz, 2H, H_2_), 5.46 (s, 2H, H_4_), 3.45 – 1.97 (m, 24H, H_6-12_), 1.48 (s, 9H, H_14_), 1.42 (s, 18H, H_13_), 1.29 (s, 18H, H_3_); **^13^C NMR** (126 MHz, CDCl_3_) δ 172.81, 172.33, 151.88, 145.36, 134.38, 122.88, 122.73, 122.04, 82.01, 56.17, 55.89, 54.72, 49.78, 48.49, 35.01, 31.52, 28.20, 28.12. Four ^13^C signals could not be found.; **HRMS** (ESI^+^): Calcd. for C_44_H_75_N_7_O_6_H^+^: 798.5852, found 798.5891 [M+H]^+^.

**4•3H**1 M NaOH (aq.) (0.33 mL, 0.33 mmol, 43 equiv.) was added to a solution of **3** (6.1 mg, 7.6 µmol, 1 equiv.) in MeOH (1.3 mL) and the mixture was left at 90 °C in a pressure tube overnight. The MeOH was removed at reduced pressure, the residue was diluted with water (5.0 mL), and pH was adjusted until neutral by slow addition of 1 M HCl (aq.). After removing the water under reduced pressure, the residue was dissolved in a minimal amount of MeOH and excess NaCl was removed using SEC (MeOH, Sephadex LH-20). This yielded **4**•3H as a colorless solid (4.6 mg, 90 %).

**^1^H NMR** (500 MHz, CD_3_OD) δ 8.22 (s, 1H, H_5_), 7.40 (s, 1H, H_1_), 7.15 (d, J = 1.8 Hz, 2H, H_2_), 5.59 (s, 2H, H_4_), 4.13 (s, 2H, H_6_), 3.87 – 2.76 (m, 22H H_7-12_), 1.29 (s, 18H, H_3_). **^13^C NMR** (126 MHz, CD_3_OD) δ 174.13, 170.44, 152.59, 142.97, 136.33, 128.35, 123.28, 123.04, 56.73, 55.64, 55.10, 52.17, 51.38, 50.27, 48.36, 35.72, 31.83.One ^13^C signal seems to be overlapping with MeOD peak.; **HRMS** (ESI^+^): Calcd. for C_32_H_51_N_7_O_6_H^+^: 630.3974, found 630.3985 [M+H]^+^.

**4•[Eu]
4**•3H (4.6 mg, 6.8 µmol, 1.0 equiv.) was dissolved in MeOH (5 mL) and Eu(OTf)_3_ (4.51 mg, 7.5 µmol, 1.1 equiv.) and Et_3_N (5.7 µL, 41 µmol, 6.0 equiv.) were added. The solution was left to stir at 60 °C for 5 days, after which the solvent was removed under reduced pressure. HEt_3_NCl and excess Eu(OTf)_3_ was removed by SEC chromatography (MeOH, Sephadex LH-20), which yielded **4**•[Eu] as a colorless solid (3.1 mg, 59 %).

**^1^H NMR** (500 MHz, CD_3_OD) δ 34.39, 31.39, 31.15, 29.06, 8.15, 7.87, 7.06, 6.66, 6.37, 4.60, 3.80, 3.64, 3.19, -0.31, -0.62, -4.08, -4.20, -4.75, -6.23, -6.66, -7.22, -8.55, -8.90, -12.26, -12.65, -14.90, -16.25, -17.39, -18.25, -19.74, -20.84. **HRMS** (ESI^+^): Calcd. for C_32_H_48_O_6_N_7_EuH^+^: 780.2951, found 780.2953 [M+H]^+^.

## S4.2. Further Experimental Procedures

**Procedure for cation addition experiments:**

Two stock solutions were prepared, of **2**•[Eu] (1.0 mM in MeOH) and cation **M** (3.0 mM in MeOH; ClO_4_ salts for Mn^2+^, Fe^2+^, Co^2+^, Ni^2+^, Cu^2+^, Zn^2+^, Li^+^, Na^+^ and Ag^+^; TsOH for H^+^). Stock solution **2**•[Eu] (30 µL) and stock solution **M** (50 µL) were diluted in a quartz cuvette with MeOH (2920 µL) for each cation.

**Procedure for quenching by Cu^2+^ addition**

Two stock solutions were prepared, of **2**•[Eu] (1.0 mM in MeOH) and Cu(ClO_4_)_2_) (3.0 mM in MeOH). Stock solution **2**•[Eu] (30 µL) was diluted in a quartz cuvette with MeOH (2970 µL), and thereafter stock solution with Cu(II) was added portionwise (1.0 µL, 1.0 µL, 3.0 µL, 2.5 µL, 2.5 µL, 5.0 µL, 5.0 µL and 30 µL).

# S5. SUPPLEMENTARY DATA

## S5.1. Screening of Conditions for AMT-CuAAC Rotaxane Formation

Since the DO3A-containing **A3** is a competing Cu-coordinator to the **A2** macrocycle, optimal synthesis conditions for the AMT-CuAAC needed to be investigated to favor the formation of rotaxane **1** over the axle byproduct **3**, as well as the dealkylated axle byproduct **3A** shown in Scheme S6 below.

Scheme S6. Conditions for formation of rotaxane 1, including byproducts 3 (free axle) and 3A (dealkylated axle).

Equivalents of macrocycle **A2** to Cu^+^ in relation to the limiting reagents **A1** and **A3** (which were always added in 1:1 stoichiometry) were varied from the standard conditions (13 mM **A1**, 13 mM **A3**, 25 mM N*^i^*PrEt in MeOH). The product ratio obtained from crude NMR measurements is displayed in Table S1 and Figure S1 below. The ratio of 1.6:1.5 **A2**:[Cu] produced the most amount of rotaxane **1** and therefore these conditions were chosen for the AMT-CuAAC reaction. We repeated the reaction three further times to confirm reproducibility (Table S2 and Figure S2). This demonstrated good reproducibility in rotaxane yield and rotaxane/axle ratio over several runs, with some minor variations.

**Reaction optimization:**

Figure S1. Product distribution of rotaxane 1 (blue), axle 3 (orange) and dealkylated axle 3A (gray) obtained from crude ^1^H-NMR spectra using different equivalents of macrocycle A2 and [Cu(MeCN)_4_][PF_6_] under standard conditions.

Table S1. Product distribution obtained from crude ^1^H-NMR analysis using different equivalents of macrocycle A2 and [Cu(MeCN)_4_][PF_6_]. Standard conditions as given in text.

| A2 : [Cu] | 1 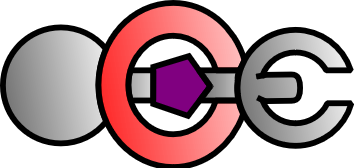 | 3  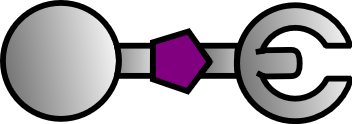 | 3A 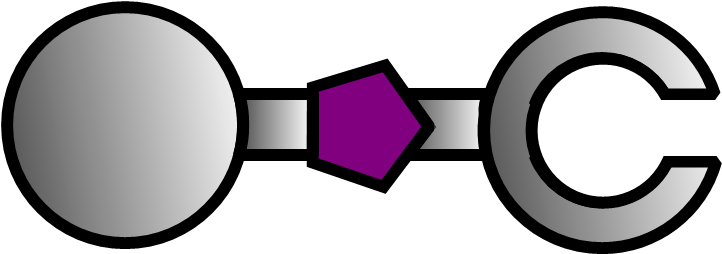 |
| --- | --- | --- | --- |
| 1.0 : 1.0 | 11 % | 81 % | 8 % |
| 1.0 : 2.2 | 27 % | 59 % | 14 % |
| 2.2 : 2.0 | 34 % | 12 % | 54 % |
| 1.6 : 1.5 | 59 % | 30 % | 12 % |

**Investigating reproducibility:**

Figure S2. Product distribution of rotaxane 1 (blue), axle 3 (orange) and dealkylated axle 3A (gray) obtained from crude ^1^H-NMR spectra when repeating the optimal conditions using 1.6 and 1.5 equivalents of A2 and [Cu(MeCN)_4_][PF_6_].

Table S2. Product distribution obtained from crude ^1^H-NMR analysis when repeating the optimal conditions using 1.6 and 1.5 equivalents of A2 and [Cu(MeCN)_4_][PF_6_]. Standard conditions as given in text.

| A2 : [Cu] | 1 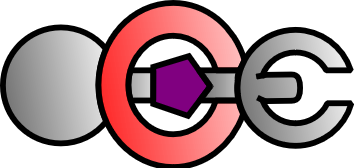 | 3  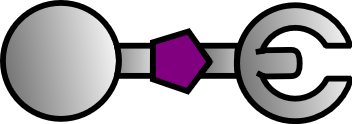 | 3A 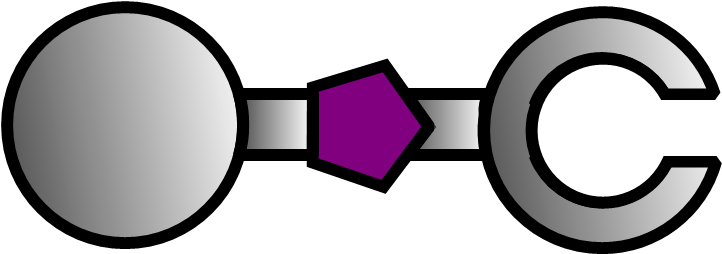 |
| --- | --- | --- | --- |
| 1.6 : 1.5 | 59 % | 30 % | 12 % |
| 1.6 : 1.5 | 52 % | 48 % | 0 % |
| 1.6 : 1.5 | 69 % | 31 % | 0 % |
| 1.6 : 1.5 | 75 % | 25 % | 0 % |

## S5.2. Stability Study of Rotaxane

To analyze long-term rotaxane integrity, a sample of rotaxane **2**•3H (the interlocked species with the smallest stopper in this study) was kept in CD_3_OD at room temperature under air for three months, before reanalysis. As can be seen in Figure S3 below, there was no sign of decomposition, indicating that the mechanical bond is stable over time.

Figure S3. ^1^H-NMR spectra (500 MHz, CD_3_OD, 298 K) of rotaxane 2•3H at synthesis (top) and after 3 months (bottom).

## S5.3. Protonation of DO3A-containing Compounds

DO3A containing compounds, such as compound **1**, are basic and can be protonated by even trace amount of acid. We routinely found that **1** got protonated even when running SiO_2_ columns or using acidic CDCl_3_ for NMR samples. We hence found that it was useful for routine purity analysis to ensure full protonation of **1**. This was achieved by titrating stoichiometric amounts of HCl into the NMR samples post-dissolution, which led to full protonation of the DO3A and greatly facilitated analysis. An example of the ^1^H-NMR spectra of rotaxane **1** before and after acid addition is presented in Figure S4 below.

**1•H^+^**

**1•H^+^**

**1**

**1•H^+^**

Figure S4. An ^1^H NMR (500 MHz, CDCl_3_, 298 K) titration of 1 using HCl in MeOH.

## S5.4. Lanthanide Coordination

To assess coordination degree of lanthanide ions to DO3A units, HPLC-MS was primarily used. By running samples containing non-coordinated rotaxane **2**•3H and coordinated rotaxane **2**•[Ln] on a C18-column using 50 % MeCN and 50 % H_2_O (with 0.1 % formic acid), baseline separation of the two compounds was observed with retention times 1.27 and 2.3 min for **2**•3H and **2**•[Eu], respectively (Figure S5). As can been seen in Figure S6 below, no trace of **2**•3H could be seen in the sample of **2**•[Eu] post-reaction, indicating full coordination.

Figure S5. HPLC traces of 2•3H (green) and 2•[Eu] (purple) with their respective retention times of 1.3 and 2.3 min using a C18-column and 50 % MeCN and 50 % H_2_O (with 0.1 % formic acid).


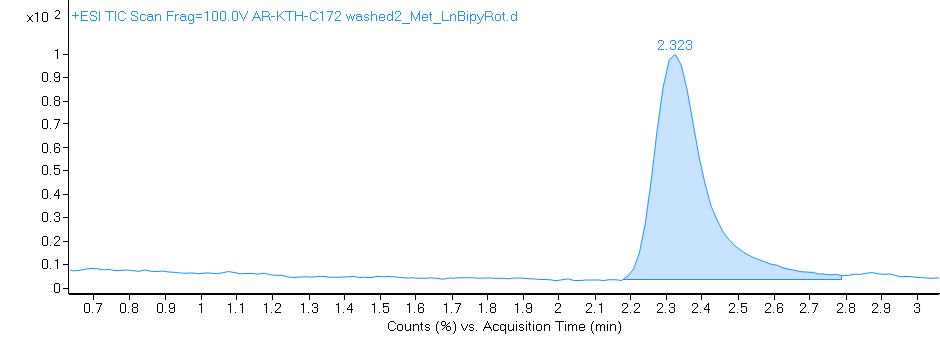


Figure S6. HPLC trace of 2•[Eu] with a retention time of 2.3 min using a C18-column and 50 % MeCN and 50 % H_2_O (with 0.1 % formic acid).

For some lanthanides, it is also possible to use NMR spectroscopy to monitor the complexation. While interpretation of paramagnetic NMR spectra is difficult, we could still compare the paramagnetically affected protons in the ^1^H-NMR spectra of **2**•[Eu] and **4**•[Eu] (Figure S7). In particular, there are diagnostic paramagnetically shifted protons around 9-13 ppm which are present in **2**•[Eu] but not in **4**•[Eu], stemming from the macrocycle (encircled area).

Figure S7. ^1^H NMR (500 MHz, CD_3_OD, 298 K) titration of rotaxane 2•[Eu] (top) and axle 4•[Eu] (bottom).

## S5.5. Photophysical Characterization

### S5.5.1. 2•[Eu]

Figure S8. Normalized absorption (gray), excitation (λ_emission_ = 616 nm, steady-state, blue) and emission spectra (λ_excitation_ = 275 nm, steady-state, red; time-gated with 0.05 ms, orange) of 2•[Eu] (MeOH, RT).

Figure S9. Absorption spectrum of 2•[Eu] (10 µM, MeOH, RT).

Figure S10. Excitation spectrum (λ_emission_ = 616 nm) of 2•[Eu] (10 µM, MeOH, RT).

Figure S11. Emission spectrum (λ_excitation_ = 275 nm, steady-state) of 2•[Eu] (10 µM, MeOH, RT).

Figure S12. Emission spectrum (λ_excitation_ = 275 nm, time-gated with 0.05 ms) of 2•[Eu] (10 µM, MeOH, RT).

Figure S13. Luminescence lifetime emission decay profile 2•[Eu] (10 µM, MeOH, RT, λ_excitation_ = 275 nm).

### S5.5.2. 2•[Tb]

Figure S14. Normalized absorption (gray), excitation (λ_emission_ = 545 nm, steady-state, blue) and emission spectra (λ_excitation_ = 275 nm, steady-state, green; time-gated with 0.05 ms, yellow) of 2•[Tb] (MeOH, RT).

Figure S15. Absorption spectrum of 2•[Tb] (10 µM, MeOH, RT).

Figure S16. Excitation spectrum (λ_emission_ = 545 nm) of 2•[Tb] (10 µM, MeOH, RT).

Figure S17. Emission spectrum (λ_excitation_ = 275 nm, steady-state) of 2•[Tb] (10 µM, MeOH, RT).

Figure S18. Emission spectrum (λ_excitation_ = 275 nm, time-gated with 0.05 ms) of 2•[Tb] (10 µM, MeOH, RT).

### S5.5.3. 4•[Eu]

Figure S19. Normalized absorption (gray), excitation (λ_emission_ = 700 nm, steady-state, blue) and emission spectra (λ_excitation_ = 262 nm, steady-state, black) of 4•[Eu] (MeOH, RT).

Figure S20. Absorption spectrum of 4•[Eu] (50 µM, MeOH, RT).

Figure S21. Excitation spectrum (λ_emission_ = 700 nm) of 4•[Eu] (20 µM, MeOH, RT).

Figure S22. Emission spectrum (λ_excitation_ = 262 nm, steady-state) of 4•[Eu] (20 µM, MeOH, RT).

### S5.5.4. q-values

The coordination environment was analyzed and compared for rotaxane **2**•[Eu] and axle **4**•[Eu] by measuring their respective emission lifetimes and calculating the corresponding q-value according to the equation below.^97^ As can be seen in Table S3, the measured q-values are almost identical, meaning that the coordination environment around Eu^3+^ is not significantly affected by the macrocycle.

**q-value**:

$$q_{Eu}=2.1\left( \frac{1}{\tau_{MeOH}}-\frac{1}{\tau_{MeOD}}-0.0 \right)$$

where *τ*_MeOH_ and *τ*_MeOD_ are observed lifetimes in MeOH and MeOD, respectively.

Table S3. Measured lifetimes of 2•[Eu] and 4•[Eu] in MeOH and MeOD along with the calculated q-value. For each lifetime τ, three repeat measurements were performed.

| Complex | *τ*_MeOH_ (ms) | *τ*_MeOD_ (ms) | q-value |
| --- | --- | --- | --- |
| 2•[Eu] | 0.993 | 2.328 | 1.2 |
| 4•[Eu] | 1.232 | 3.610 | 1.1 |

## S5.6. Emission Amplification via Intercomponent Energy Transfer

To quantify the sensitization effect of the macrocycle on the lanthanide emission, the intensity at the respective excitation maxima of the rotaxane **2**•[Eu] and axle **4**•[Eu] was measured (see Figure S23). The excitation maximum for the emission peak at 700 nm is 274 nm and 262 nm for **2**•[Eu] and **4**•[Eu], respectively (see Figure S24). The emission amplification at the different excitation wavelengths is presented in Table S4 below. An additional excitation wavelength of 280 nm was also chosen to show that the macrocycle in **2**•[Eu] can sensitize at higher wavelengths, resulting in a 37-fold increase compared to the trizole unit in **4**•[Eu].

Table S4. Relative emission intensity of rotaxane 2•[Eu] and axle 4•[Eu] at different excitation wavelengths, where 262 and 274 nm is the excitation maximum of 4•[Eu] and 2•[Eu], respectively (20 µM, MeOH, RT).

| Excitation wavelength (2•[Eu]/4•[Eu]) | 262/262 nm | 274/262 nm | 280/280 nm |
| --- | --- | --- | --- |
| Max. emission intensity 2•[Eu] (a.u.) | 43957 | 44929 | 40690 |
| Max. emission intensity 4•[Eu] (a.u.) | 6564 | 6564 | 1111 |
| Relative intensity (2•[Eu]/4•[Eu]) | 6.7 | 6.8 | 37 |

Figure S23. Emission spectrum (20 µM, steady state, MeOH, RT) of 2•[Eu] at λ_excitation_ = 274 nm (red), λ_excitation_ = 280 nm (blue) and λ_excitation_ = 262 nm (green), as well as 4•[Eu] at λ_excitation_ = 280 nm (blue, dashed) and λ_excitation_ = 262 nm (green, dashed).

Figure S24. Excitation spectrum at λ_emission_ = 700 nm (20 µM, steady state, MeOH, RT) of 2•[Eu] (red) and 4•[Eu] (blue).

## S5.7. Metal Addition to 2•[Eu]

We also investigated the coordination of metals and other cations to **2•**[Eu] by direct addition. If a cation coordinates to the bipyridine nitrogen atoms in **2**•[Eu], a redshift of the absorption spectrum is expected (see main manuscript).^78^

### S5.7.1. Absorption Spectra

The samples were prepared according to the general procedure in section S4.2, where 5 equivalents of the perchlorate metal salts were added to a solution of **2**•[Eu] (For TsOH 1 equivalent was added as Eu-DOTA coordination is sensitive to excess acid). When assessing the shape of the absorption spectra in Figure S26 and Figure S28, it seems that Cu^2+^, Fe^2+^ and Ag^+^ have some binding affinity to **2**•[Eu].

Figure S25. Absorption spectrum of 2•[Eu] (10 µM, MeOH, RT, black) with 5 equiv. of Mn(ClO_4_)_2_ (orange), Zn(ClO_4_)_2_ (gray), Ni(ClO_4_)_2_ (yellow), Cu(ClO_4_)_2_ (red), Co(ClO_4_)_2_ (green) and Fe(ClO_4_)_2_ (blue). The spectra have been baseline adjusted.

Figure S26. Normalized absorption spectrum of 2•[Eu] (10 µM, MeOH, RT, black) with 5 equiv. of Mn(ClO_4_)_2_ (orange), Zn(ClO_4_)_2_ (gray), Ni(ClO_4_)_2_ (yellow), Cu(ClO_4_)_2_ (red), Co(ClO_4_)_2_ (green) and Fe(ClO_4_)_2_ (blue).

Figure S27. Absorption spectrum of 2•[Eu] (10 µM, MeOH, RT, black) with 1 equiv. of TsOH (yellow) and 5 equiv. of LiClO_4_ (blue), NaClO_4_ (green) and AgClO_4_ (red). The spectra have been baseline adjusted.

Figure S28. Normalized absorption spectrum of 2•[Eu] (10 µM, MeOH, RT, black) with 1 equiv. of TsOH (yellow) and 5 equiv. of LiClO_4_ (blue), NaClO_4_ (green) and AgClO_4_ (red).

### S5.7.2. Emission Spectra

The samples were prepared according to the general procedure in section S4.2, where 5 equivalents of the perchlorate metal salts were added to a solution of **2**•[Eu] (1 equiv. for TsOH).

When comparing the intensity of the Eu-emission, one can compare different wavelengths. Below is Figure S29, where the intensities from the emission maxima at 615 nm and 700 nm are compared for all metals. This is summarized in Table S5, as well. The raw data comparisons are given in Figure S30 – Figure S39, below.

Figure S29. Relative emission intensities (λ_excitation_ = 275 nm, steady-state) of of 2•[Eu] (10 µM, MeOH, RT, black) with 5 equiv. of metals (exception TsOH with 1 equiv.) at λ_emission_ of 615 nm (black) and 700 nm (blue).

Table S5. Relative emission intensities (λ_excitation_ = 275 nm, steady-state) of of 2•[Eu] (10 µM, MeOH, RT, black) with 5 equiv. of metals (exception TsOH with 1 equiv.) at λ_emission_ of 615 nm and 700 nm.

|  | 615 nm (%) | 700 nm (%) |
| --- | --- | --- |
| 2•[Eu] | 100 | 100 |
| Zn^2+^ | 86 | 107 |
| Cu^2+^ | 4.5 | 5.2 |
| Ni^2+^ | 82 | 93 |
| Co^2+^ | 61 | 73 |
| Fe^2+^ | 64 | 83 |
| Mn^2+^ | 57 | 67 |
| H^+^ | 57 | 69 |
| Li^+^ | 82 | 100 |
| Na^+^ | 93 | 112 |
| Ag^+^ | 69 | 73 |

Figure S30. Emission spectrum (λ_excitation_ = 275 nm, steady-state) of 2•[Eu] (10 µM, MeOH, RT, black) and after addition of 5 equiv. of Zn(ClO_4_)_2_ (gray).

Figure S31. Emission spectrum (λ_excitation_ = 275 nm, steady-state) of 2•[Eu] (10 µM, MeOH, RT, black) and after addition of 5 equiv. of Cu(ClO_4_)_2_ (blue).

Figure S32. Emission spectrum (λ_excitation_ = 275 nm, steady-state) of 2•[Eu] (10 µM, MeOH, RT, black) and after addition of 5 equiv. of Ni(ClO_4_)_2_ (yellow).

Figure S33. Emission spectrum (λ_excitation_ = 275 nm, steady-state) of 2•[Eu] (10 µM, MeOH, RT, black) and after addition of 5 equiv. of Co(ClO_4_)_2_ (green).

Figure S34. Emission spectrum (λ_excitation_ = 275 nm, steady-state) of 2•[Eu] (10 µM, MeOH, RT, black) and after addition of 5 equiv. of Fe(ClO_4_)_2_ (blue).

Figure S35. Emission spectrum (λ_excitation_ = 275 nm, steady-state) of 2•[Eu] (10 µM, MeOH, RT, black) and after addition of 5 equiv. of Mn(ClO_4_)_2_ (orange).

Figure S36. Emission spectrum (λ_excitation_ = 275 nm, steady-state) of 2•[Eu] (10 µM, MeOH, RT, black) and after addition of 1 equiv. of TsOH (pink).

Figure S37. Emission spectrum (λ_excitation_ = 275 nm, steady-state) of 2•[Eu] (10 µM, MeOH, RT, black) and after addition of 5 equiv. of LiClO_4_ (blue).

Figure S38. Emission spectrum (λ_excitation_ = 275 nm, steady-state) of 2•[Eu] (10 µM, MeOH, RT, black) and after addition of 5 equiv. of NaClO_4_ (green).

Figure S39. Emission spectrum (λ_excitation_ = 275 nm, steady-state) of 2•[Eu] (10 µM, MeOH, RT, black) and after addition of 5 equiv. of AgClO_4_ (red).

### S5.7.3. Quenching Titration of 2•[Eu] with Cu^2+^

The quenching titration in Figure S40 below was performed according to general procedure in section S4.2, where up to 5 equivalents of Cu(ClO_4_)_2_ were added. To confirm the integrity of the rotaxane under these conditions, we also checked for presence of **2**•[Eu] (and absence of **2**•[Cu]) with HPLC-MS after adding 5 equivalents of copper. No Cu-complex was observed and **2**•[Eu] was entirely intact, meaning that the copper does not displace the europium and is weakly bound to the assembly.

Figure S40. Emission spectra (λ_excitation_ = 275 nm, steady-state) of 2•[Eu] (10 µM, MeOH, RT, black) with different equivalents of Cu(ClO_4_)_2_.

### S5.7.4. q-values

Since redshifted absorption spectra were observed upon addition of some cations to **2**•[Eu], it was of interest to investigate whether the coordination sphere around the lanthanide changed during these coordination events. Comparing the values in Table S6 below, one can see that the q-value does not change significantly for any of the investigated coordinating cations, which means that the coordination sphere is not significantly affected by the addition of these metals. The q-value for Cu^2+^ could not be accurately recorded due to the Eu-emission being too low at these concentrations, leading to a low apparent lifetime.

**q value**:^97^

$$q_{Eu}=2.1\left( \frac{1}{\tau_{MeOH}}-\frac{1}{\tau_{MeOD}}-0.0 \right)$$

where *τ*_MeOH_ and *τ*_MeOD_ are observed lifetimes in MeOH and MeOD, respectively.

Table S6. Measured lifetimes of 2•[Eu], as well as with different metals (5 equiv. for Cu, Fe, Ag and 1 equiv. for H) in MeOH and MeOD along with the calculated q-value. For each lifetime τ, three repeat measurements were performed.

| Complex | *τ*_MeOH_ (ms) | *τ*_MeOD_ (ms) | q-value |
| --- | --- | --- | --- |
| 2•[Eu] | 0.993 | 2.328 | 1.21 |
| 2•[Eu] + Fe^2+^ | 0.976 | 2.339 | 1.25 |
| 2•[Eu] + H^+^ | 0.998 | 2.254 | 1.17 |
| 2•[Eu] + Ag^+^ | 0.942 | 1.988 | 1.17 |

# **S6. COMPUTATIONAL DETAILS**

## **S6.1. Geometry Optimization**

Initial geometry optimization was carried out on **2**•[Eu] using xTB software (version 6.7.0)^81,98^ using the semi-empirical quantum mechanical (SQM) method GFN2-xTB.^99^ These were further reoptimized at the same level of theory in methanol with implicit solvent, using the ALPB solvation model.^83^ These geometries were further optimized using density functional theory (DFT) calculations which were performed using the Orca 6.0.1 package,^100^ and methanol as implicit solvent (CPCM solvation model). Lanthanide atoms were described with a polarized valence triple-zeta basis set (def2-TZVP), loading a Stuttgart-Dresden effective core potential (def2-ECP, core size = 28) for Eu, and light atoms were described with a polarized split-valence basis set (def2-SVP).^85^ Firstly, DFT optimizations were carried out using the BP86 exchange-correlation functional,^101,102^ employing Fermi-smearing^103^ (1500 K) and replacing the Eu atom with La, to assist with convergence of the spin-unrestricted self-consistent field (SCF). This optimized **2**•[La] structure was then further optimized at the same level of theory with Eu using broken-symmetry DFT,^104^ followed by optimization and Hessian calculations with the ωB97X-D3 functional.^86^ All geometries of optimized minima are provided as xyz coordinates in an additional supplementary .zip file.

## **S6.2. Conformational Sampling**

Conformational sampling of the GFN2-xTB optimized **2**•[Eu] was carried out using the default metadynamics-based iMTD-GC workflow of Grimme’s *C*onformer-*R*otamer *E*nsemble *S*ampling *T*ool *CREST* (version 3.0.2)^84^ at the GFN2-xTB level of theory using methanol as implicit solvent (ALPB solvation model).^83^ After sorting with the CREGEN function of CREST, the resulting ensemble contained 240 conformers with energies within 6 kcal mol^-1^ of the global minimum, and 17 conformers within 1.5 kcal mol^-1^ from the global minimum. Due to the large size and flexibility of the system, these low-lying conformations had multiple different variations of similar structural motifs. As such, the ensemble was investigated manually and from the 17 conformers (<1.5 kcal mol^-1^ away from the global minimum), three representative structures (STRUC1, STRUC5 and STRUC14) were selected to demonstrate the different conformations of the rotaxane, illustrating pirouetting of the macrocycle around the thread (Table S7). All CREST energies (crest.energies) and conformers (crest_conformers.xyz) as well as specific xyz coordinates for STRUC1, STRUC5 and STRUC14 can be found in an additional supplementary file (.zip).

Table S7. Select GFN2-xTB optimized structures (ALPB methanol) from CREST conformational sampling with energies <1.5 kcal mol^-1^. Left picture shows a side view, right picture shows a top-down view.

| Name | GFN2-xTB optimized structure | | Relative energy (kcal mol^-1^) | Eu–N Distance (Å) |
| --- | --- | --- | --- | --- |
| STRUC1 (global minimum) | 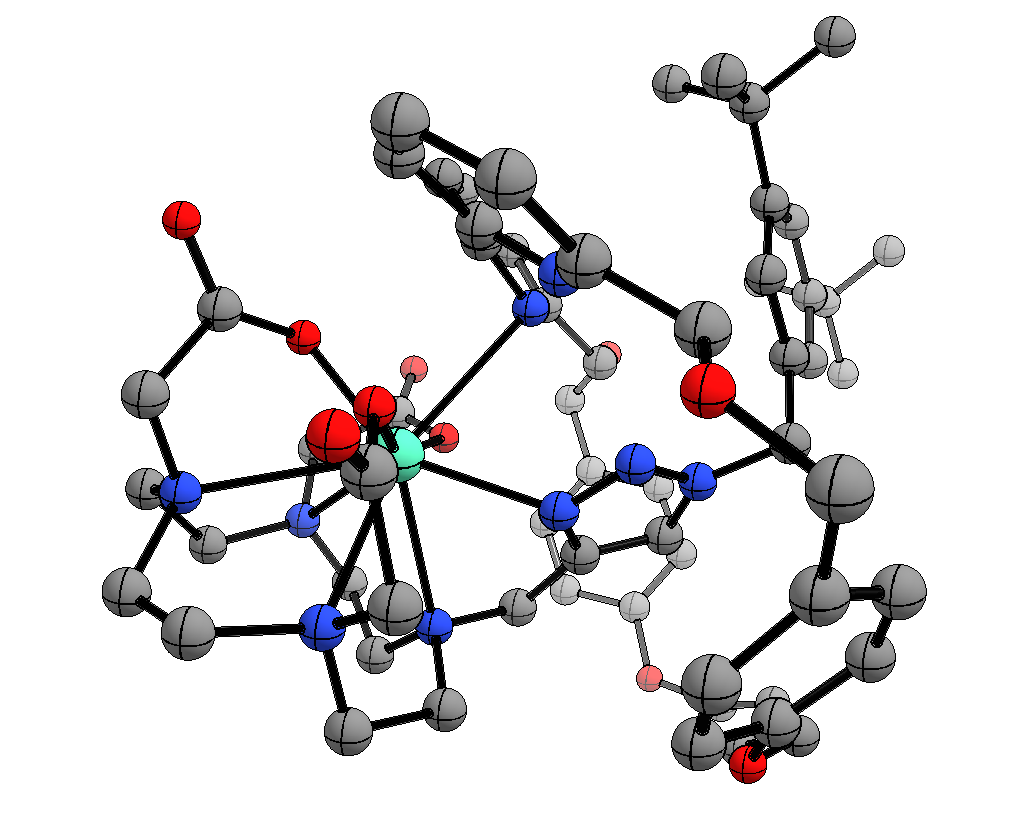 | 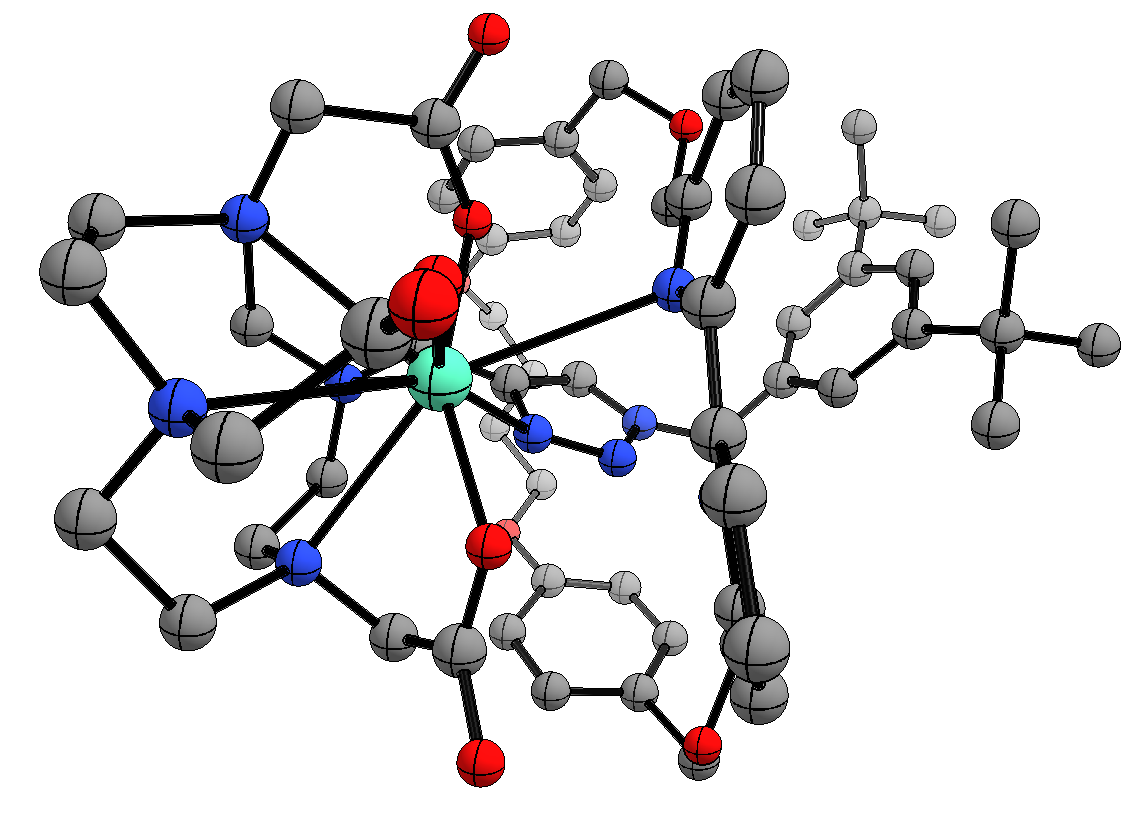 | 0 | 3.07 |
| STRUC5  (Bipy away from Eu) | 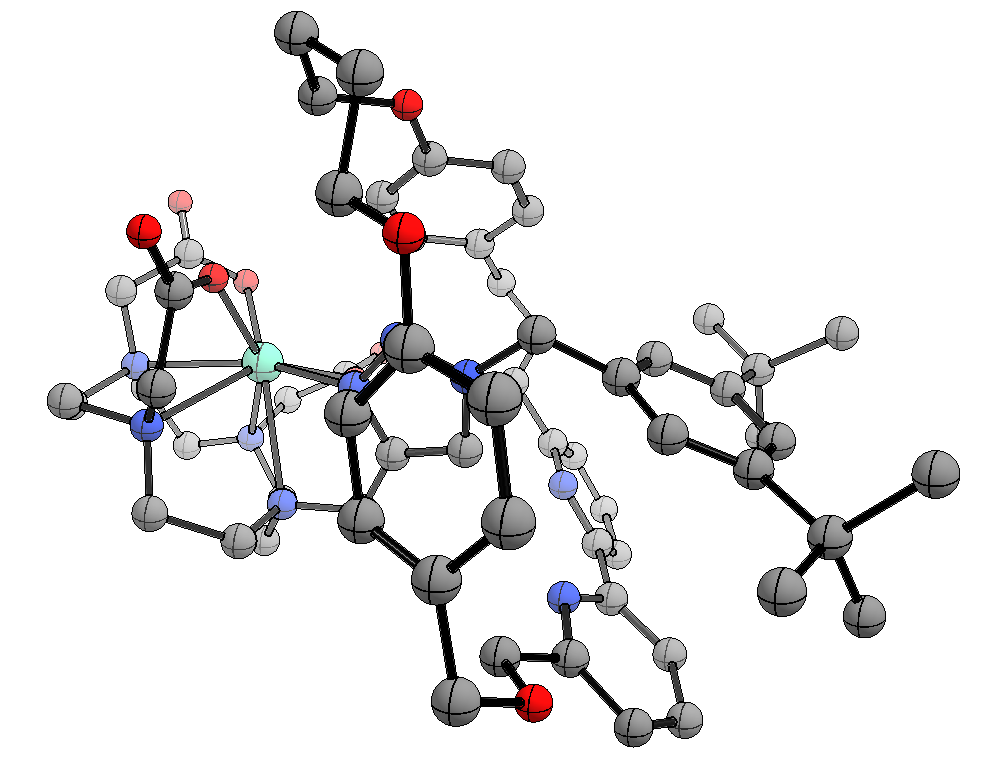 | 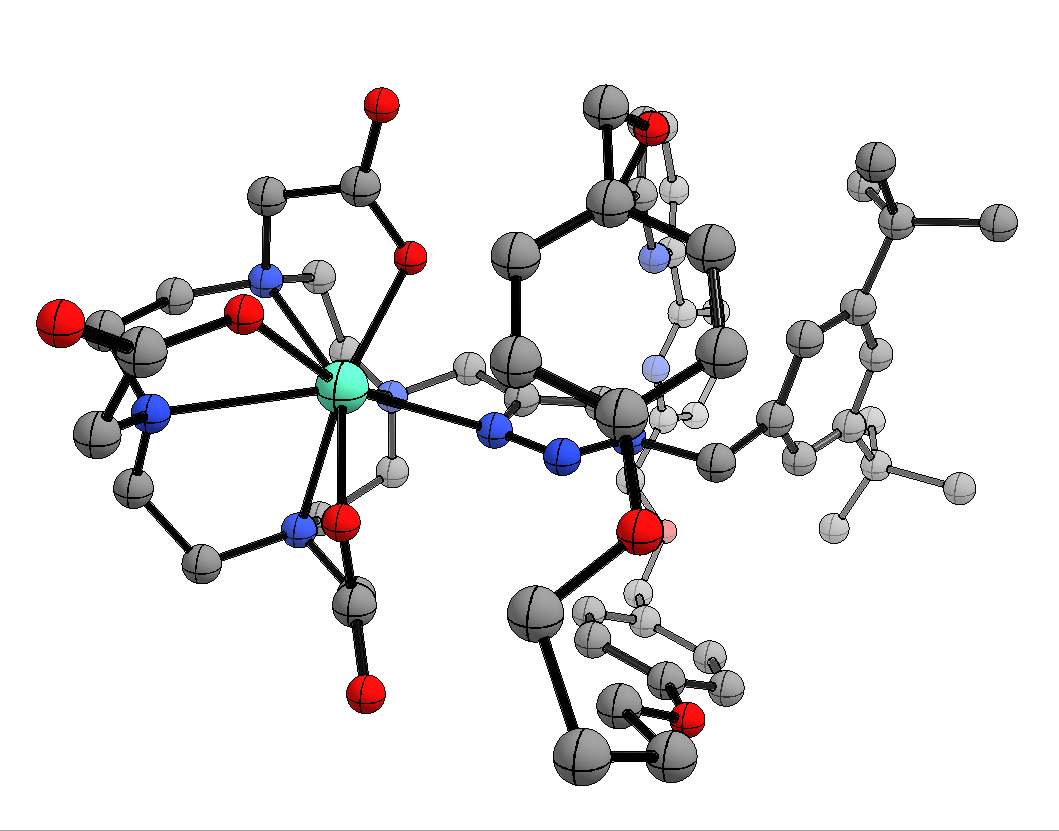 | 1.09 | 7.06 |
| STRUC14  (Bipy close to Eu) | 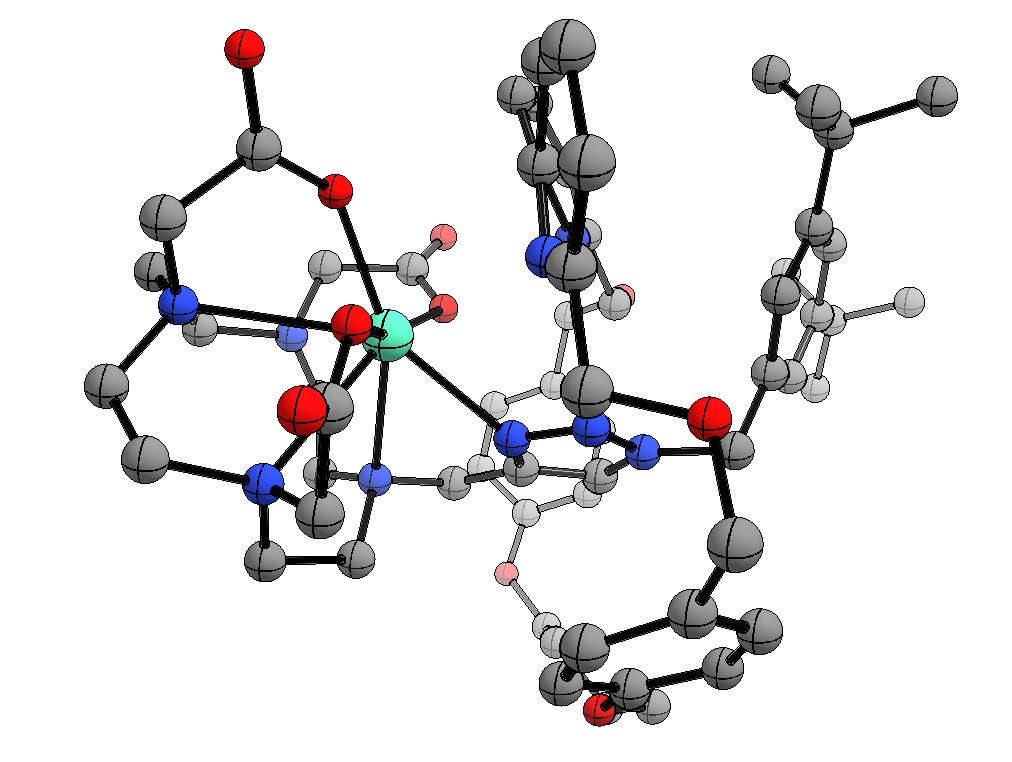 | 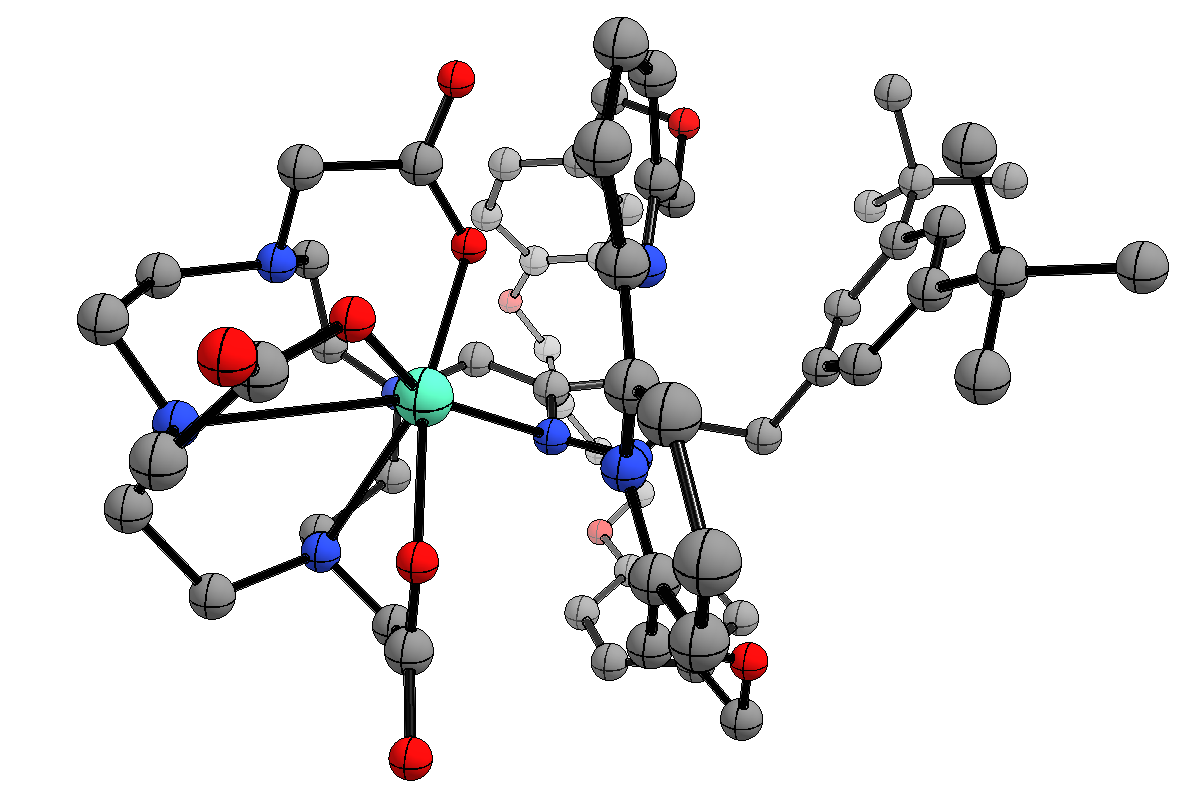 | 1.39 | 3.38 |

## **S6.3. Metadynamics Simulations**

To probe the pirouetting mechanism of the macrocycle about the thread of **2**•[Eu], biased molecular dynamics (metadynamics) simulations^88^ were employed at the GFN2-xTB level of theory with methanol as implicit solvent (ALPB solvation model). The total length of the metadynamics simulation was 1 ns, using the root mean square deviation (RMSD) of all atoms as the collective variable. The hydrogen mass was set to 4 amu, and covalent bonds were maintained using the SHAKE algorithm. The simulation parameters included a temperature of 298 K, a time step of 4 fs, k_push_ of 0.1, and an α of 1.2 Bohr^-1^. The resulting trajectories were visualized and analyzed using ChemCraft.

## **S6.4. Space-filling Models of** 2•[Eu]

To visualize how the macrocycle completely shields the lanthanide ion, space-filling representations of the DFT-optimized structure of **2**•[Eu] at the ωB97X-D3/def2-SVP(Eu, def2-TZVP)/CPCM(MeOH)//GFN2-xTB/ALPB(MeOH) level of theory are presented from three angles (Figure S41). Lanthanide ion (in green) is not visible through any angle.

**
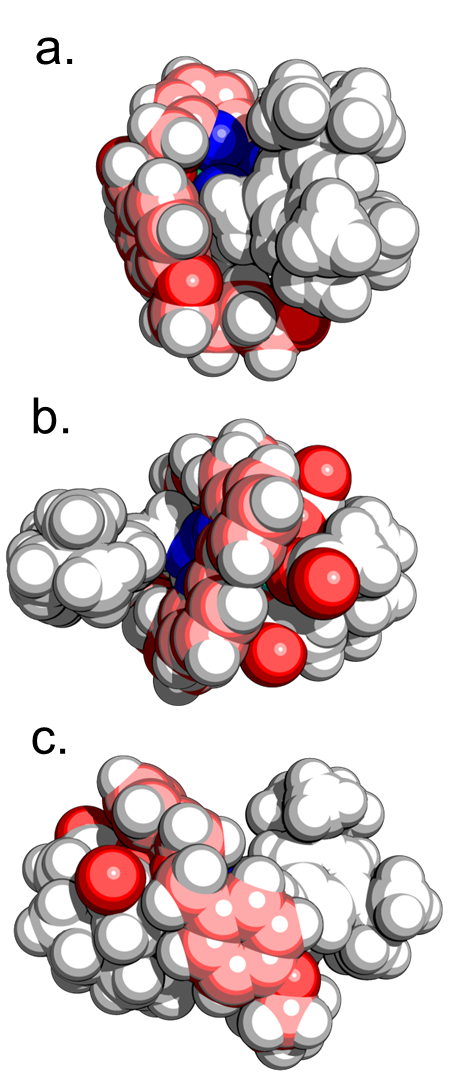
**

Figure S41. Space-filling models of the DFT-optimised structure of 2•[Eu]. a) Viewed along axle; b) tilted side view; c) straight side view.

## **S6.5. Explicit solvation of** 2•[Eu] using Quantum Cluster Growth

To probe the effect of explicit solvation on the metadynamics^88^ of **2**•[Eu], the Quantum Cluster Growth (QCG) method from Grimme was employed^[89]^ using CREST (version 3.0.2) and xTB (version 6.6.1).^[[2]](#footnote-3)^ Solvent shells of various sizes (n-solvation = 12, 40) of explicit methanol molecules were built around **2**•[Eu], followed by short runs of metadynamics (50 ps, with the same settings described in S6.3.) to probe the co-conformational dynamics of **2**•[Eu]. No significant difference to the co-conformational dynamics was found between explicit and implicit solvation models, showing similar Eu–N distances throughout. All xyz coordinates for the various QCG solvent clusters as well as the input co-ordinates for the solute (**2**•[Eu]) and solvent (methanol) can be found in an additional supplementary file (.zip).

S7. MASS SPECTRA


Figure S42. High-resolution ESI-MS (positive mode) of 1 (top: full spectrum; bottom: zoom, with predicted isotope pattern in red).

Figure S43. High-resolution ESI-MS (positive mode) of 2•3H (top: full spectrum; bottom: zoom, with predicted isotope pattern in red).

Figure S44. High-resolution ESI-MS (positive mode) of 2•[Eu] (top: full spectrum; bottom: zoom, with predicted isotope pattern in red).

Figure S45. High-resolution ESI-MS (positive mode) of 2•[Lu] (top: full spectrum; bottom: zoom, with predicted isotope pattern in red).

Figure S46. High-resolution ESI-MS (positive mode) of 2•[Gd] (top: full spectrum; bottom: zoom, with predicted isotope pattern in red).

Figure S47. High-resolution ESI-MS (positive mode) of 2•[Tb] (top: full spectrum; bottom: zoom, with predicted isotope pattern in red).

Figure S48. High-resolution ESI-MS (positive mode) of 3 (top: full spectrum; bottom: zoom, with predicted isotope pattern in red).

Figure S49. High-resolution ESI-MS (positive mode) of 4•3H (top: full spectrum; bottom: zoom, with predicted isotope pattern prediction in red).

Figure S50. High-resolution ESI-MS (positive mode) of 4•[Eu] (top: full spectrum; bottom: zoom, with predicted isotope pattern in red).

# S8. NMR SPECTRA

Spectrum S1. ^1^H NMR spectrum (500 MHz, CDCl_3_, 298 K) of 1.

Spectrum S2. ^13^C NMR spectrum (126 MHz, CDCl_3_, 298 K) of 1.

Spectrum S3. COSY NMR spectrum (500 MHz, CDCl_3_, 298 K) of 1.

Spectrum S4. DEPT NMR spectrum (126 MHz, CDCl_3_, 298 K) of 1.

Spectrum S5. ^1^H-^13^C HSQC NMR spectrum (500 MHz, CDCl_3_, 298 K) of 1.

Spectrum S6. ^1^H-^13^C HMBC NMR spectrum (500 MHz, CDCl_3_, 298 K) of 1.

Spectrum S7. ^1^H NMR spectrum (500 MHz, CD_3_OD, 298 K) of 2•3H.

Spectrum S8. ^13^C NMR spectrum (126 MHz, CD_3_OD, 298 K) of 2•3H.

Spectrum S9. COSY NMR spectrum (500 MHz, CD_3_OD, 298 K) of 2•3H.

Spectrum S10. ^1^H-^13^C HSQC NMR spectrum (500 MHz, CD_3_OD, 298 K) of 2•3H.

Spectrum S11. ^1^H-^13^C HMBC NMR spectrum (500 MHz, CD_3_OD, 298 K) of 2•3H.

Spectrum S12. ^1^H NMR spectrum (500 MHz, CD_3_OD, 298 K) of 2•[Eu].

Spectrum S13. ^1^H NMR spectrum (400 MHz, CD_3_OD, 298 K) of 2•[Tb].

Spectrum S14. ^1^H NMR spectrum (500 MHz, CD_3_OD, 298 K) of 2•[Gd].

Spectrum S15. ^1^H NMR spectrum (500 MHz, CD_3_OD, 298 K) of 2•[Lu].

Spectrum S16. ^1^H NMR spectrum (500 MHz, CDCl_3_, 298 K) of 3.

Spectrum S17. ^13^C NMR spectrum (126 MHz, CDCl_3_, 298 K) of 3.

Spectrum S18. COSY NMR spectrum (500 MHz, CDCl_3_, 298 K) of 3.

Spectrum S19. ^1^H-^13^C HSQC NMR spectrum (500 MHz, CDCl_3_, 298 K) of 3.

Spectrum S20. ^1^H NMR spectrum (500 MHz, CD_3_OD, 298 K) of 4•3H.

Spectrum S21. ^13^C NMR spectrum (126 MHz, CD_3_OD, 298 K) of 4•3H.

Spectrum S22. COSY NMR spectrum (500 MHz, CD_3_OD, 298 K) of 4•3H.

Spectrum S23. DEPT NMR spectrum (126 MHz, CD_3_OD, 298 K) of 4•3H.

Spectrum S24. ^1^H-^13^C HSQC NMR spectrum (500 MHz, CD_3_OD, 298 K) of 4•3H.

Spectrum S25. ^1^H-^13^C HMBC NMR spectrum (500 MHz, CD_3_OD, 298 K) of 4•3H.

Spectrum S26. ^1^H NMR spectrum (500 MHz, CD_3_OD, 298 K) of 4•[Eu].

# S9. REFERENCES

For references [71], [78], [81], [83-85], [88], and [89] see main manuscript.

[94] E. A. Neal, S. M. Goldup, A Kinetic Self-Sorting Approach to Heterocircuit [3]Rotaxanes, *Angew. Chem. Int. Ed.* **2016**, *55*, 12488–12493; *Angew. Chem.* **2016**, *128*, 12676–12681.

[95] D. A. Moore, Selective Trialkylation of Cyclen with *tert*-Butyl Bromoacetate, *Org. Synth*. **2008**, *85*, 10.

[96] A. K. R. Junker, M. Tropiano, S. Faulkner, T. J. Sørensen, Kinetically Inert Lanthanide Complexes as Reporter Groups for Binding of Potassium by 18-Crown-6, *Inorg. Chem*. **2016**, *55*, 12299–12308.

[97] R. C. Holz, C. A. Chang, W. D. Horrocks Jr., Spectroscopic Characterization of the Europium(III) Complexes of a Series of N,N’-Bis(Carboxymethyl) Macrocyclic Ether Bis(Lactones), *Inorg. Chem.* **1991**, *30*, 3270–3275.

[98] C. Bannwarth, E. Caldeweyher, S. Ehlert, A. Hansen, P. Pracht, J. Seibert, S. Spicher, S. Grimme, Extended Tight-Binding Quantum Chemistry Methods. *Wiley Interdiscip. Rev. Comput. Mol. Sci.* **2021**, *11*, e1493.

[99] C. Bannwarth, S. Ehlert, S. Grimme, GFN2-xTB—An Accurate and Broadly Parametrized Self-Consistent Tight-Binding Quantum Chemical Method with Multipole Electrostatics and Density-Dependent Dispersion Contributions. *J. Chem. Theory Comput.* **2019**, *15*, 1652–1671.

[100] F. Neese, Software Update: The ORCA Program System—Version 5.0. *Wiley Interdiscip. Rev. Comput. Mol. Sci.* **2022**, *12*, e1606.

[101] A. D. Becke, Density-Functional Exchange-Energy Approximation with Correct Asymptotic Behavior. *Phys. Rev. A* **1988**, *38*, 3098–3100.

[102] J. P. Perdew, Density-Functional Approximation for the Correlation Energy of the Inhomogeneous Electron Gas, *Phys. Rev. B,* **1986**, *33*, 8822–8824.

[103] S. Grimme, A. Hansen, A Practicable Real-Space Measure and Visualization of Static Electron-Correlation Effects, *Angew. Chem. Int. Ed.* **2015**, *54*, 12308–12313.

[104] F. Neese, Definition of Corresponding Orbitals and the Diradical Character in Broken Symmetry DFT Calculations on Spin Coupled Systems, *J. Phys. Chem. Solids,* **2004**, *65*, 781–785.

1. Lanthanide coordination was tracked by HPLC-MS. Note that the rotaxanes complex much faster than the free axle, which needs heating to 60 ºC and extended reaction time. The origin of this difference is not clear to us at the moment. [↑](#footnote-ref-2)
2. An older version of xTB was used due to errors with the 6.7.0 version building the QCG sphere. See issue #1080 on the Grimme xTB/CREST github: <https://github.com/grimme-lab/xtb/issues/1080> (accessed 30^th^ April 2025) [↑](#footnote-ref-3)
